# The PPARγ‐SETD8 axis constitutes an epigenetic, p53‐independent checkpoint on p21‐mediated cellular senescence

**DOI:** 10.1111/acel.12607

**Published:** 2017-05-17

**Authors:** Chieh‐Tien Shih, Yi‐Feng Chang, Yi‐Tung Chen, Chung‐Pei Ma, Hui‐Wen Chen, Chang‐Ching Yang, Juu‐Chin Lu, Yau‐Sheng Tsai, Hua‐Chien Chen, Bertrand Chin‐Ming Tan

**Affiliations:** ^1^ Graduate Institute of Biomedical Sciences College of Medicine Chang Gung University Kwei‐San, Tao‐Yuan Taiwan; ^2^ Molecular Medicine Research Center Chang Gung University Tao‐Yuan Taiwan; ^3^ Department of Biomedical Sciences College of Medicine Chang Gung University Kwei‐San, Tao‐Yuan Taiwan; ^4^ Department of Physiology and Pharmacology College of Medicine Chang Gung University Kwei‐San, Tao‐Yuan Taiwan; ^5^ Division of Endocrinology and Metabolism Department of Internal Medicine Chang Gung Memorial Hospital Linkou, Tao‐Yuan Taiwan; ^6^ Institute of Clinical Medicine National Cheng Kung University Tainan Taiwan; ^7^ Department of Neurosurgery Lin‐Kou Medical Center Chang Gung Memorial Hospital Linkou, Tao‐Yuan Taiwan

**Keywords:** cellular senescence, epigenetic, H4K20me1, p21, p53, SETD8/KMT5A

## Abstract

Cellular senescence is a permanent proliferative arrest triggered by genome instability or aberrant growth stresses, acting as a protective or even tumor‐suppressive mechanism. While several key aspects of gene regulation have been known to program this cessation of cell growth, the involvement of the epigenetic regulation has just emerged but remains largely unresolved. Using a systems approach that is based on targeted gene profiling, we uncovered known and novel chromatin modifiers with putative link to the senescent state of the cells. Among these, we identified SETD8 as a new target as well as a key regulator of the cellular senescence signaling. Knockdown of SETD8 triggered senescence induction in proliferative culture, irrespectively of the p53 status of the cells; ectopic expression of this epigenetic writer alleviated the extent doxorubicin‐induced cellular senescence. This repressive effect of SETD8 in senescence was mediated by directly maintaining the silencing mark H4K20me1 at the locus of the senescence switch gene *p21*. Further in support of this regulatory link, depletion of p21 reversed this SETD8‐mediated cellular senescence. Additionally, we found that PPARγ acts upstream and regulates *SETD8* expression in proliferating cells. Downregulation of PPARγ coincided with the senescence induction, while its activation inhibited the progression of this process. Viewed together, our findings delineated a new epigenetic pathway through which the PPARγ‐SETD8 axis directly silences *p21* expression and consequently impinges on its senescence‐inducing function. This implies that SETD8 may be part of a cell proliferation checkpoint mechanism and has important implications in antitumor therapeutics.

## Introduction

The process of biological aging is described as senescence, through which the function of a mature organism gradually deteriorates, ultimately leading to mortality. One aspect of the senescence is ‘cellular senescence’, which was initially observed in cultured fibroblasts that, upon finite number of cell cycle, irreversibly cease to proliferate—a state also called replicative senescence or the Hayflick limit. In addition to this replicative senescence that is attributed to telomere shortening (d'Adda di Fagagna *et al*., 2003), senescence could also be induced by DNA‐damaging agents or aberrant oncogene activation, thereby acting as a protective response (Halazonetis *et al*., 2008; Gorgoulis & Halazonetis, 2010). Given this presumably tumor‐suppressive nature, senescence is viewed as a barrier to cancer and therefore a potential basis for therapeutic regimen (Collado & Serrano, 2010; Nardella *et al*., 2011).

While the senescent cells are dormant in terms of growth, they remain metabolically active and are characterized by distinct molecular and cellular changes. Aside from a permanent cell cycle arrest, there could be morphological transformation, activation of tumor suppressor networks associated with the p53 and p16(INK4A)‐RB signal cascades, increased activity of the lysosomal β‐D‐galactosidase (SA‐β‐GAL), altered chromatin structure (senescence‐associated heterochromatic foci or SAHF) and prominent changes in secretomes such as cytokines and chemokines—the so‐called senescence‐associated secretory phenotype (SASP) (Kuilman *et al*., 2010; Nardella *et al*., 2011). Mechanistically, several key aspects of gene regulation have been known to underlie this programmed cessation of cell growth, such as such as transcriptional alteration (Jing & Lee, 2014; Hesp *et al*., 2015) and miRNA‐mediated post‐transcriptional silencing (Lafferty‐Whyte *et al*., 2009). Recently, a role of the epigenetic regulation in cellular senescence has also emerged—DNA and histone modifiers, such as DNA methyltransferase (DNMT1) (Cruickshanks *et al*., 2013), EZH2 (Fan *et al*., 2011), Jarid1a, and Jarid1b (Chicas *et al*., 2012), have been found to impact the progression of this process. However, the functional relevance of this chromatin‐based mechanism has not been characterized in depth.

Methylation of histone H4 at lysine 20 (H4K20me) is one of the central epigenetic marks, with extensive implications in fundamental processes such as DNA damage repair, chromatin compaction, and DNA replication (Beck *et al*., 2012). The methyltransferases SETD8 catalyzes the initial mono‐methylation, upon which SUV4‐20H1 and SUV4‐20H2 enzymes mediate further modification to H4K20me2 and H4K20me3, respectively (Beck *et al*., 2012). Conversely, removal of the methyl moiety is carried out by a group of ‘erasers’ – PHF8 and PHF2, which, respectively, catalyze demethylation of H4K20me1 and H4K20me3 (Liu *et al*., 2010; Stender *et al*., 2012). Finally, the functional output of this modification type may be transduced by the L3MBTL1 protein, which is a MBT domains‐containing transcription repressor that binds to both mono‐ and di‐methylated H4K20 (Trojer *et al*., 2007; West *et al*., 2010).

In mammals, SETD8 is one of the methyltransferases that target both histones and nonhistone proteins. Aside from the side‐chain amino group of H4K20, SETD8 also executes mono‐methylation of proliferating cell nuclear antigen (PCNA) at lysine 248 (Takawa *et al*., 2012) and of p53/TP53 at lysine 382 (Shi *et al*., 2007). Through these enzymatic activities, SETD8 has been found to play multiple roles in DNA damage response, mitotic condensation and DNA replication (Beck *et al*., 2012): Lys248 methylation of PCNA stabilizes the protein, therefore promoting the proliferation of cancer cells (Takawa *et al*., 2012). Moreover, by virtue of interacting with PCNA and creating the H4K20me1 mark for 53BP1 binding, SETD8 is linked to the safeguarding of genome duplication and integrity (Oda *et al*., 2010). SETD8 also suppresses p53 transactivation activity and consequently the downstream apoptosis function, by the direct modification of Lys382 (Shi *et al*., 2007). Interestingly, despite this connection with p53, SETD8 has been found to mediate several related processes independently of this tumor suppressor (Houston *et al*., 2008).

In line with the above functional attributes, several lines of evidence have ascribed a pro‐tumorigenic role to SETD8: (i) Overexpression of SETD8 is detected in various types of cancer (Takawa *et al*., 2012). (ii) Conversely, reduced SETD8 expression is associated with a better survival rate in multiple cancer types, including small‐cell lung cancer, ovarian cancer, hepatocellular carcinoma, and breast cancer (Milite *et al*., 2016). This reduction in expression and tumor susceptibility is attributed to a single‐nucleotide polymorphism in the *SETD8* gene known to alter miRNA targeting of the transcribed product. (iii) Finally, SETD8 also controls tumor metastatic potential by promoting TWIST‐dependent epithelial–mesenchymal transition (EMT) (Yang *et al*., 2012b). Considered together, SETD8 and the H4K20me mark serve as critical determinant in the control of cell proliferation in both physiological and patho‐physiological contexts.

To further elucidate the contribution of epigenetic mechanism to the cellular senescence process, we designed and performed a targeted expression screen. Based on this systems’ approach, we identified SETD8 as one of the altered epigenetic modifiers during doxorubicin‐induced cellular senescence. Drug‐induced or siRNA‐mediated downregulation of SETD8 closely correlated with transition of cells into a senescence state, characterized by morphological changes and marker expression. Intriguingly, this negative regulatory role of SETD8 in senescence was independent of p53 but by means of a direct epigenetic silencing of the *p21* gene. We further discovered that transcription factor PPARγ acts upstream of SETD8 and maintains its expression in the proliferating cells as well as its antisenescence function. In summary, our results uncovered a PPARγ‐SETD8 regulatory axis that impinges on the *p21*‐driven senescence, and further underscored a new important cellular role of the epigenetic writer SETD8 that may be mechanistic linked to its pro‐tumorigenic function.

## Results

### Identification of epigenetic components underlying the doxorubicin‐induced cellular senescence process

**Figure 1:**
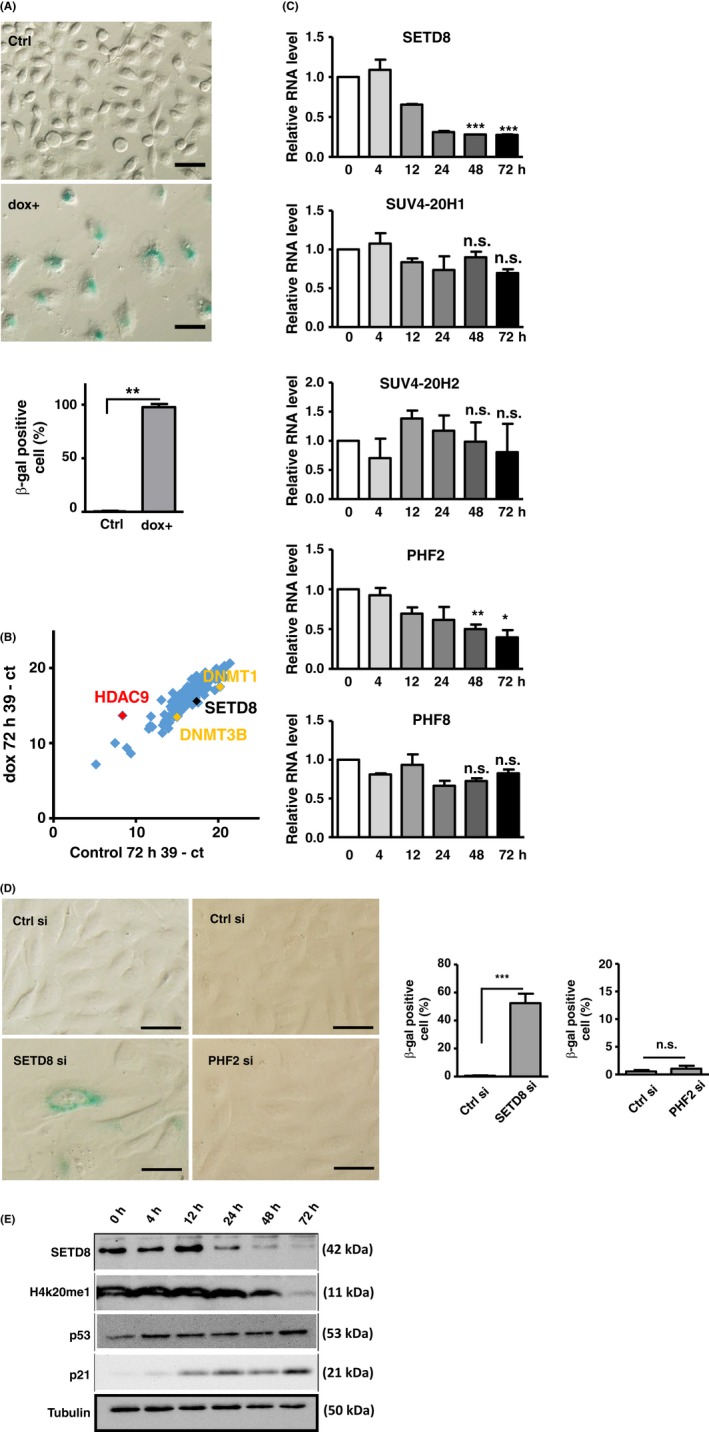
A targeted epigene expression screen uncovers alteration of histone H4K20 methylation modifiers during doxorubicin (dox)‐induced cellular senescence. (A) As the basis of the screen, OC3 cells were treated with low concentration of dox (50 nm) for 3 days to induce cellular senescence, as revealed by β‐gal‐positive staining (Scale bar, 50 μm). Bar graph on the bottom shows the proportion of β‐gal‐positive cells. (B) The cells from (A) were then subjected to gene expression profiling as outlined in the Experimental procedures section. The scatter plot profiles the relative expression levels (Ct values) of epigene targets in the control (*x*‐axis) vs. dox‐treated (*y*‐axis) culture. Genes previously implicated in the senescence process are marked; SETD8 denotes a new target and the focus of this study. (C) Validation of the senescence‐associated expression profiles was done by real‐time RT–PCR. OC3 cells were harvested from control (0 h) or dox treatment (4, 12, 24, 48, 72 h) conditions. Expression patterns for modifiers of the H4K20 mark were selectively shown, with data being normalized to the levels at 0 h. (D) U2OS cells were depleted of SETD8 or PHF2 expression by RNAi and subsequently monitored for the senescence‐associated β‐gal staining. Bar graphs on the right represent the quantified results (*n* > 100) of the microscopy data shown on the left (scale bar, 50 μm). (E) Upon senescence induction as in (C), cell extracts were prepared for immunoblot analysis of SETD8, H4K20me1, p53, and p21. Tubulin serves as the internal control. For experiments shown in Fig. 1, all quantitative measurements are presented as mean ± SE of at least three independent experiments (n.s.: not significant; *P < 0.05; **P < 0.01; ***P < 0.001).

Given the emerging link of epigenetic regulation to the cellular senescence pathway, we first set out to systematically profile the senescence‐associated expression of known epigenetic regulators as a means to delineate further the underlying mechanism. For this purpose, a total of 135 epigenetic modulators were manually selected, for which we designed specific primers for real‐time PCR experiments (see Experimental procedures). An *in vitro* senescence model was established by subjecting OC3 cells to a 3‐days doxorubicin (dox) treatment (at 50 nm) (Chang *et al*., 1999). OC3 was chosen as the model owing to its availability and responsiveness to dox‐induced senescence. This treatment elicited DNA damage (Fig. S1A) and largely recapitulated the hallmarks of cellular senescence, such as β‐gal‐positive staining, SASP expression, cell cycle arrest at the G2/M phase, and altered expression of cell cycle‐related genes (Fig. 1A and Fig. S1B–F). We then analyzed the temporal expression patterns of the preselected 135 epigenetic factors (Fig. 1B) and subsequently found that 17 genes underwent upregulation (*P* < 0.05, ≧ 2‐fold change; Table S1) while 26 genes with downregulation (*P* < 0.05, ≦ 2‐fold change; Table S2) in response to the induction of cellular senescence. As proof of principle, several genes previously found altered during the course of senescence were also identified in our screen—DNMT1 and DNMT3B were similarly downregulated (Dodge *et al*., 2005; Cruickshanks *et al*., 2013), whereas a known senescence marker HDAC9 was upregulated (Mason *et al*., 2004). Interestingly, SETD8, a histone H4K20 methyltransferase, underwent a noticeable reduction in expression in the dox‐induced, senescent cell culture (Fig. 1C).

### Epigenetic modifiers of H4K20 methylation were altered during doxorubicin‐induced cellular senescence

While a role of SETD8 in cell senescence has not been hitherto reported, previous studies have hinted a link of H4K20 methylation to this process (Chicas *et al*., 2012) and reported a reduction in the levels of this marker in older senescence‐accelerated mouse prone 8 (Wang *et al*., 2010). To next confirm our results on SETD8 and to further examine possible senescence‐associated alterations of other epigenetic modulators of H4K20 methylation during dox‐induced cellular senescence, we performed real‐time PCR experiments to independently measure the relative expression of H4K20 methyltransferases (SETD8, SUV4‐20H1, and SUV4‐20H2) and demethylases (PHF2 and PHF8). Our results subsequently showed that the mRNAs of both SETD8 and PHF2 underwent downregulation in the dox‐treated OC3 cells (Fig. 1C). We further examined their functional relevance by performing siRNA‐mediated knockdown experiments in the U2OS cells, which was a classical model for demonstrating the role of SETD8 in cell apoptosis (Shi *et al*., 2007). We found that depletion of PHF2 had no discernable effect on senescence‐associated cellular changes, such as β‐gal stain positivity (Fig. 1D), cell growth delay (Fig. S2A), and nuclear area enlargement (Fig. S2B). By contrast, depletion of SETD8 could induce β‐gal staining (Fig. 1D). Furthermore, in line with the alterations at the RNA level, we also found that protein expression of SETD8 gradually diminished in the course of doxorubicin‐induced cellular senescence, as shown by both immunoblotting (Fig. 1E) and immunofluorescence analyses (Fig. S3). Concordantly, the levels of H4K20me1 marker exhibited a discernable decrease under prolonged dox treatment (Figs 1E and S3).

### SETD8 is a negative regulator of the cellular senescence process

**Figure 2:**
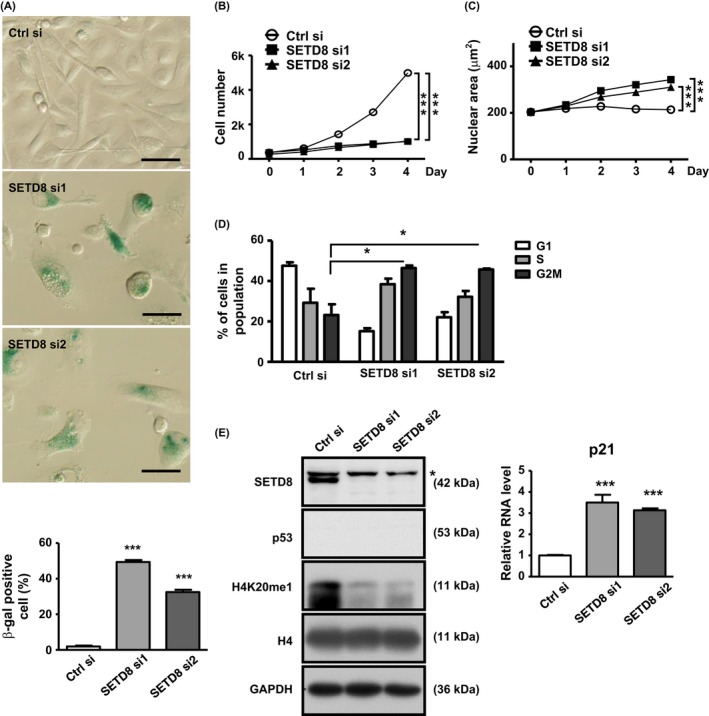
Knockdown of SETD8 triggers premature cellular senescence. (A) to (E) PC3 cells were transfected with control (Ctrl) or SETD8‐targeting siRNAs (si1 and si2) and then subjected to senescence‐related phenotype analyses (see Experimental procedures): β‐gal staining (scale bar = 50 μm for the images, with bar graph below depicting quantitative results) (A), cell proliferation curve (B), nuclear area determination (C), flow cytometry‐based cell cycle profiling (D), and *p21* transcript abundance (E). For (E), expression levels of SETD8 and H4K20me1 were verified by immunoblotting, whereas *p21* levels were determined by RT‐qPCR (normalized to *GAPDH*). The asterisk denotes a nonspecific signal in the anti‐SETD8 blot. All quantitative results shown in Fig. 2 are presented as mean ± SE of at least three independent experiments (n.s.: not significant; *P < 0.05; **P < 0.01; ***P < 0.001).

Previously, knockdown of SETD8 in U2OS cells was found to trigger cell cycle arrest and enlarged morphology (Jorgensen *et al*., 2007), which are changes reminiscent of senescence induction (Kuilman *et al*., 2010). We then set out to further strengthen the functional link of SETD8 to cellular senescence. To this end, we conducted knockdown experiments by using independent SETD8‐targeting siRNAs. This loss of SETD8 consistently led to senescence‐associated phenotypes in multiple cell lines. In PC3 cells harboring SETD8‐specific siRNAs, we detected a greater extent of β‐gal‐positive staining (Fig. 2A), cell proliferation arrest (Fig. 2B), nuclear area enlargement (Fig. 2C), G2/M arrest (Fig. 2D), and *p21* upregulation (Fig. 2E), as compared with the control cells. The downregulation of SETD8 was also corroborated by the decline in the expression of H4K20me1 marks (Fig. 2E). Further, we were able to recapitulate these phenotypes in a separate line, the U2OS cells (Fig. S4). In contrast to p21, the expression of another key senescence mediator, p16 (CDKN2A/INK4A), was either barely expressed in the U2OS cells (data not shown), or unaltered PC3 cells treated with dox or depleted of SETD8 (Fig. S5). Finally, while a role in apoptosis regulation was previously ascribed to SETD8 (Shi *et al*., 2007), our results did not show significant alterations in the apoptotic states of the SETD8 knockdown culture (Fig. S6), strongly suggesting that SETD8 may control distinct fates of the cells.

Parallel to the knockdown experiments, gain‐of‐function studies were also carried out to verify the senescence role of SETD8 (Fig. S7). Overexpression of SETD8 and the consequent upregulation of H4K20me1 were confirmed by immunoblotting (Fig. S7A). Consistent with its known pro‐proliferation role, overexpression of SETD8 in the absence of dox treatment elevated the rate of cell growth (Fig. S7B). In dox‐treated cultures undergoing senescence, introduction of ectopic SETD8 lessened cellular senescence induction to a large extent, as evidenced by the reversal of β‐gal‐positive staining (Fig. S7C), cell proliferation arrest (Fig. S7D), and upregulation of senescence markers p21, IL‐6, and IL‐8 (Fig. S7E).

### SETD8 governs suppression of senescence and associated *p21* upregulation independently of p53

Given that SETD8 is directly implicated in the Lys382 methylation of p53 and consequent modulation of its *p21*‐inducing and antiapoptotic activity (Shi *et al*., 2007), it is plausible that SETD8's action in the senescence pathway is exerted via a p53‐dependent manner. However, while depletion of SETD8 triggered premature senescence in both PC3 (Fig. 2) and U2OS (Fig. S4) cells, only the U2OS line expressed wild‐type p53, while PC3 cells lacked this protein (Figs 2E and S4E). This observation then supported the notion that SETD8 impinges on senescence independently of the p53. Moreover, the consequently altered expression of *p21*, a known downstream transcriptional target of p53, was observed in both cells, implying that *p21* may be upregulated in senescence irrespectively of p53.

**Figure 3:**
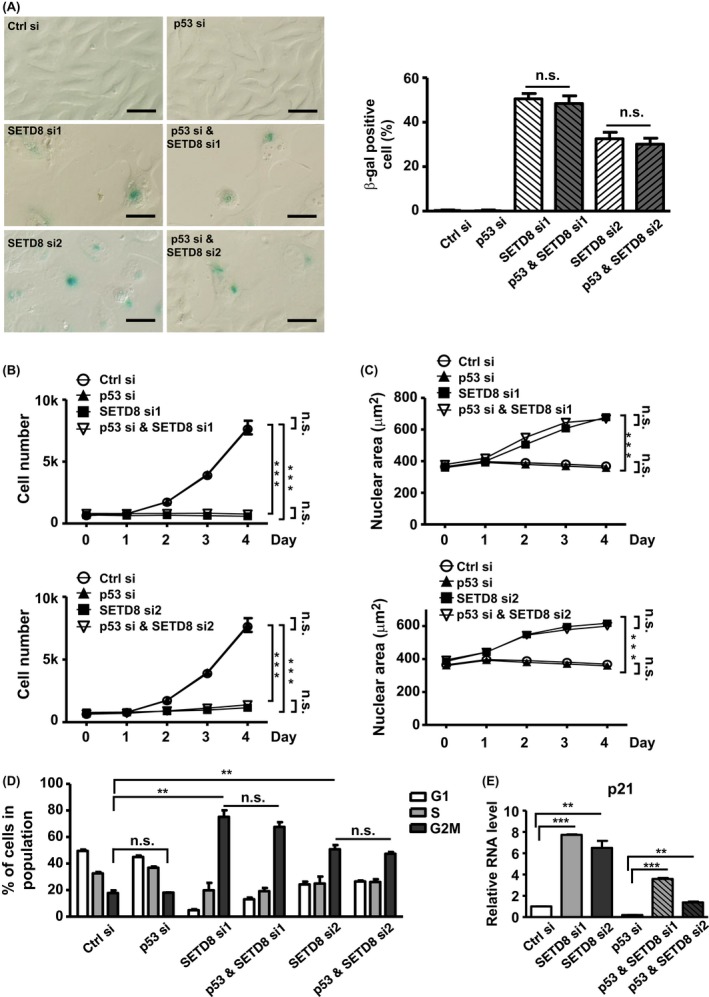
SETD8 governs cellular senescence independently of p53. (A) to (E) The U2OS cells (p53‐wt) were transfected simultaneously with SETD8 and p53 siRNAs for 6 days, and subsequently analyzed for senescence‐related phenotype as in Fig. 2: β‐gal staining (scale bar = 50 μm) (A), cell proliferation curve (B), nuclear area determination (C), flow cytometry‐based cell cycle profiling (D), and *p21* transcript abundance (E). All quantitative results shown in Fig. 3 correspond to mean ± SE of at least three independent experiments (n.s.: not significant; *P < 0.05; **P < 0.01; ***P < 0.001).

To further corroborate this p53‐independent function of SETD8 in senescence, we next performed co‐knockdown of p53 and SETD8 in the U2OS cells (Fig. S8). Interestingly, while SETD8 knockdown expectedly triggered cellular senescence, concurrent depletion of p53 did not affect this phenotype in terms of β‐gal‐positive staining (Fig. 3A), cell proliferation arrest (Fig. 3B), and nuclear area enlargement (Fig. 3C), G2/M arrest (Fig. 3D). In terms of *p21* expression, although silencing of p53 in the SETD8 knockdown cells considerably downregulated its levels, *p21* upregulation was still prominently detected in these co‐knockdown cells as compared to the p53 knockdown only group, suggesting a p53‐independent regulation (Fig. 3E). Collectively, these results served as strong evidence that, distinctively from its role in apoptosis, SETD8 hinders senescence in a p53‐independent manner.

### Depletion of p21 rescued SETD8‐mediated cellular senescence

Although p53 was dispensable to the establishment of senescence state, the upregulation of *p21* was evident in our experimental system. Through knockdown experiments, we further demonstrated the significance of p21 in conferring the proper response to senescence signaling (i.e., β‐gal‐positive staining), regardless of the p53 status (Fig. S9). These data were thus consistent with the notion that p21 is a key mediator of cellular senescence (Fang *et al*., 1999). By contrast, p21 may be dispensable to the maintenance of the senescence state—when both PC3 and U2OS cell lines were subjected to dox treatment for 7 days, cellular changes associated with senescence, such as β‐gal staining and cell cycle distribution, remained stable despite removal of dox from the culture or silencing of the *p21* gene (Fig. S10).

**Figure 4:**
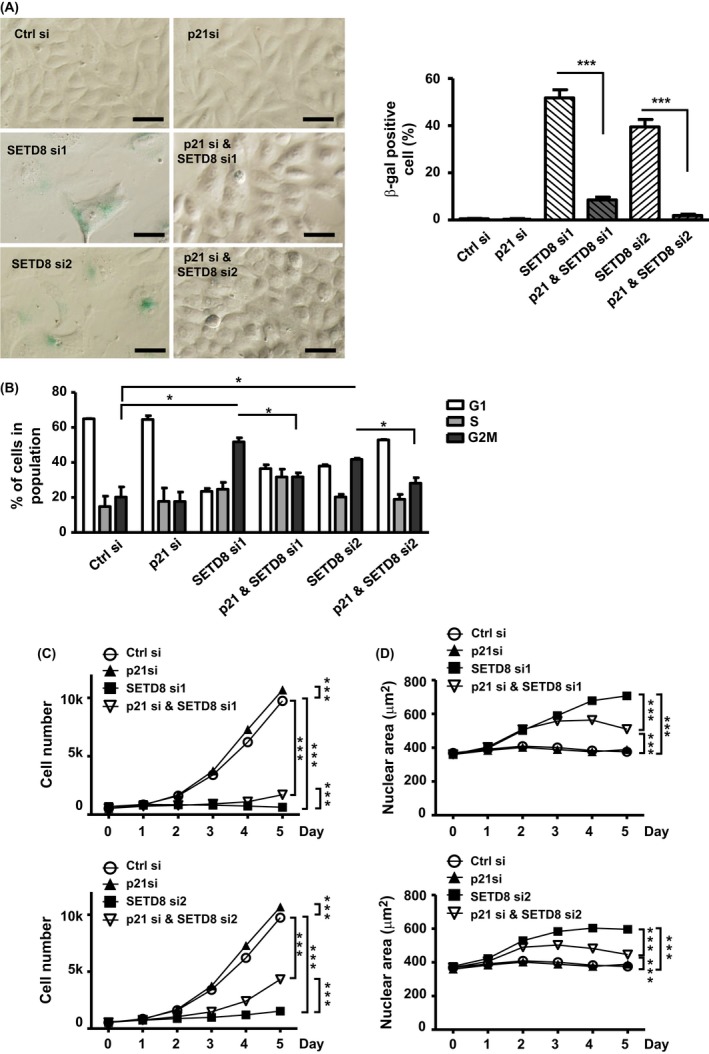
Knockdown of p21 alleviates the senescent state of the SETD8‐depleted cells. (A) to (E) U2OS cells were transfected with SETD8‐targeting siRNAs (si1 and si2), p21‐targeting siRNA, or both for 6 days. Senescence‐related characterization was then conducted as above, on the basis of β‐gal staining (scale bar = 50 μm) (A), flow cytometry‐based cell cycle profiling (B), cell proliferation rate (C), and nuclear area measurement (D). All results are shown as mean ± SE of at least three independent experiments (n.s.: not significant; *P < 0.05; **P < 0.01; ***P < 0.001).

Having demonstrated that mis‐expression of SETD8 altered *p21* expression (Figs 2E and S4E), we next set out to interrogate whether p21 underlies SETD8's antisenescence function, using co‐knockdown experiments (Fig. S11). Although RNAi‐mediated abrogation SETD8 in cycling cell expectedly induced cellular senescence, based on the proportions of β‐gal‐positive cells (Fig. 4A) and G2/M cells (Fig. 4B), simultaneous depletion of p21 notably suppressed these phenotypes. Further in line with this possible functional antagonism, concurrent depletion of p21 and SETD8 also lessened the progressive cell proliferation stall (Fig. 4C) and nuclei enlargement (Fig. 4D), as compared with cells with downregulated SETD8 only. Similar observations on this interaction were made in the PC3 cell culture (Figs S11 and S12). Considered together, these results strongly supported the physiological significance of a SETD8‐p21 network in cellular senescence.

### H4K20me1 marker association with p21 promoter region in cellular senescence was regulated by SETD8

**Figure 5:**
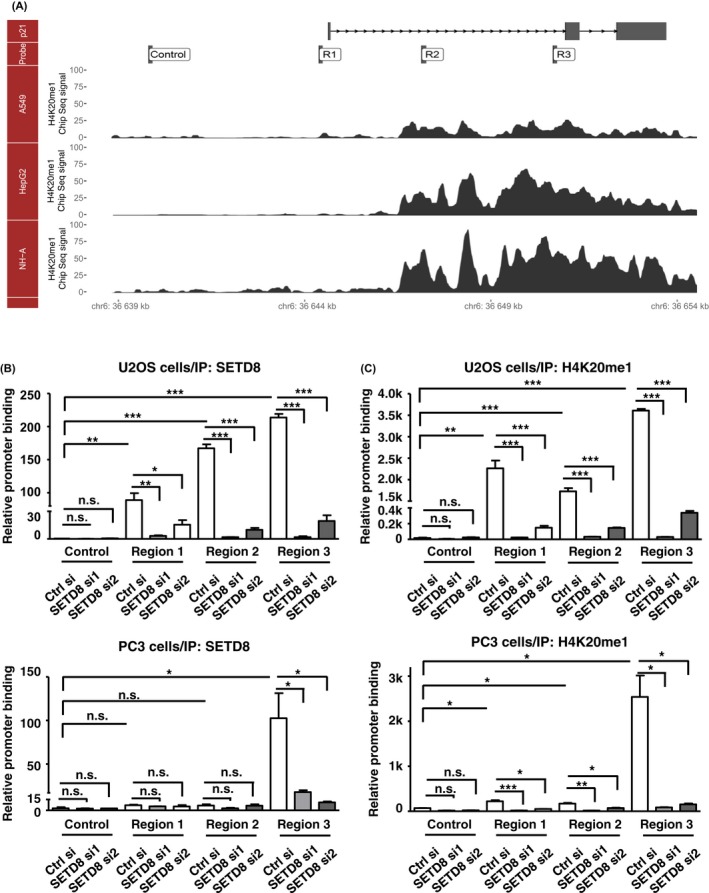
SETD8 mediates epigenetic silencing of the *p21* gene locus. (A) Schematic drawing of the *p21* gene structure and its genome location. Relative positions of the primers designed for the ChIP assay are depicted below (in the Probe track): control, R1, R2, and R3. In addition, positional occupancy of the H4K20me1 mark in the indicated cell lines, as annotated in the ENCODE data, is adapted from the UCSC genome browser and represented in chromatogram traces at the bottom. (B, C) Chromatin association of SETD8 and the H4K20me1 mark with the *p21* promoter region. U2OS and PC3 cells were transfected with control (Ctrl) or SETD8 siRNAs for 3 days. ChIP analysis was done on chromatin isolated from these cells, using control (IgG), SETD8 or H4K20me‐1 antibodies. The precipitated DNA fragments were quantitatively analyzed by real‐time PCR using the indicated primer pairs, and normalized to the values of IgG. Control primers serve as nonbinding control. Statistical significance of the indicated comparisons: n.s.: not significant; *P < 0.05; **P < 0.01; ***P < 0.001.

To clarify the molecular basis of SETD8's regulation of *p21* expression, we next sought to examine the promoter chromatin association of this epigenetic repressor. To this end, we employed the ChIP assay and designed several primer pairs for amplifying distinct regions of the *p21* gene locus (Fig. 5A). In both U2OS and PC3 cells, we were able to observe significant occupancy of SETD8 in the *p21* gene locus, with a preferential distribution in the intragenic region (Fig. 5B). The specificity of our ChIP observations was reinforced by the absence of fragments corresponding to an upstream ‘Control’ region in the anti‐SETD8 immunoprecipitates, as well as by the prominent reduction in the extent of chromatin binding detected in the SETD8 knockdown cells (Fig. 5B). While previous studies showed that H4K20me1 is undetectable in the promoter region (Shi *et al*., 2007), our ChIP results indicated that the presence of SETD8 is enriched in the gene body region (regions 2 and 3). Interestingly, the association of the downstream H4K20me mark with *p21* locus as revealed by ChIP assay was in strong correspondence to the SETD8 binding patterns (Fig. 5C). In addition, RNAi depletion of SETD8 effectively abolished this regional occupancy of H4K20me, further signifying a functional association of SETD8's activity with *p21* expression regulation. However, while ectopic expression of SETD8 also altered the levels of *IL‐6* and *IL‐8* (Fig. S7E), SETD8 was not detectable in the promoter regions of these gene loci (Fig. S13). Overall, this series of ChIP data implied that, by directly associating with the *p21* gene locus, SETD8 acts to maintain a silenced epigenetic state of this senescence switch gene. This regulation may in turn confer a cellular state unsusceptible to senescence induction.

### PPARγ acts upstream of SETD8 and maintains its expression and antisenescence function

We next sought to identify molecule(s) that acts upstream of the senescence‐associated downregulation of SETD8. Given that SETD8 is reportedly under the control of microRNAs (miRNAs) (Milite *et al*., 2016), we first searched for putative 3′ UTR‐targeting miRNAs through bioinformatics means (TargetScan) (Fig. S14A). We next profiled the expression of these candidate miRNAs in the presence of dox treatment by RT‐qPCR and further found three miRNAs—microRNA‐26a, microRNA‐29a, and microRNA‐194—that exhibited upregulation and thus could presumably contrast the expression of SETD8 during senescence induction (Fig. S14B). However, the expression of a known SETD8‐targeting miRNA, miRNA‐502, did not change in the course of dox treatment (Fig. S14B). To further implicate these miRNAs in the regulation of SETD8, and consequently the process the senescence, we next characterized the effect of ectopically expressing these miRNAs. While overexpression of ectopic miRNAs diminished the levels of endogenous *SETD8* mRNA transcripts (data not shown), it lacked inhibitory effect on a *SETD8*‐3′ UTR reporter expression (Fig. S14C,D). Moreover, we found that their overexpression in the cells did not affect several features of the senescence process (Fig. S14E–G). Therefore, these results together failed to substantiate the link of these selected miRNAs to the SETD8‐associated senescence pathway.

We next set out to assess whether the expression changes of SETD8 in response to senescence could be attributed to transcriptional regulation. In this regard, we tested possible involvement of two transcription factors, c‐MYC and PPARγ, which were previously demonstrated to mediate the expression of *SETD8* (Wakabayashi *et al*., 2009; Driskell *et al*., 2012; Ke *et al*., 2014). These two factors were chosen also due to their presumed roles in the senescence of various cancer cell lines (Wu *et al*., 2007; Briganti *et al*., 2014). As opposed to the expression of SETD8, levels of c‐MYC underwent a dramatic increase in response to senescence (Fig. S15A). Moreover, ectopic overexpression of c‐MYC did not influence the expression of SETD8 mRNA (Fig. S15B), likely suggesting that it is not directly involved in this functional regard.

**Figure 6:**
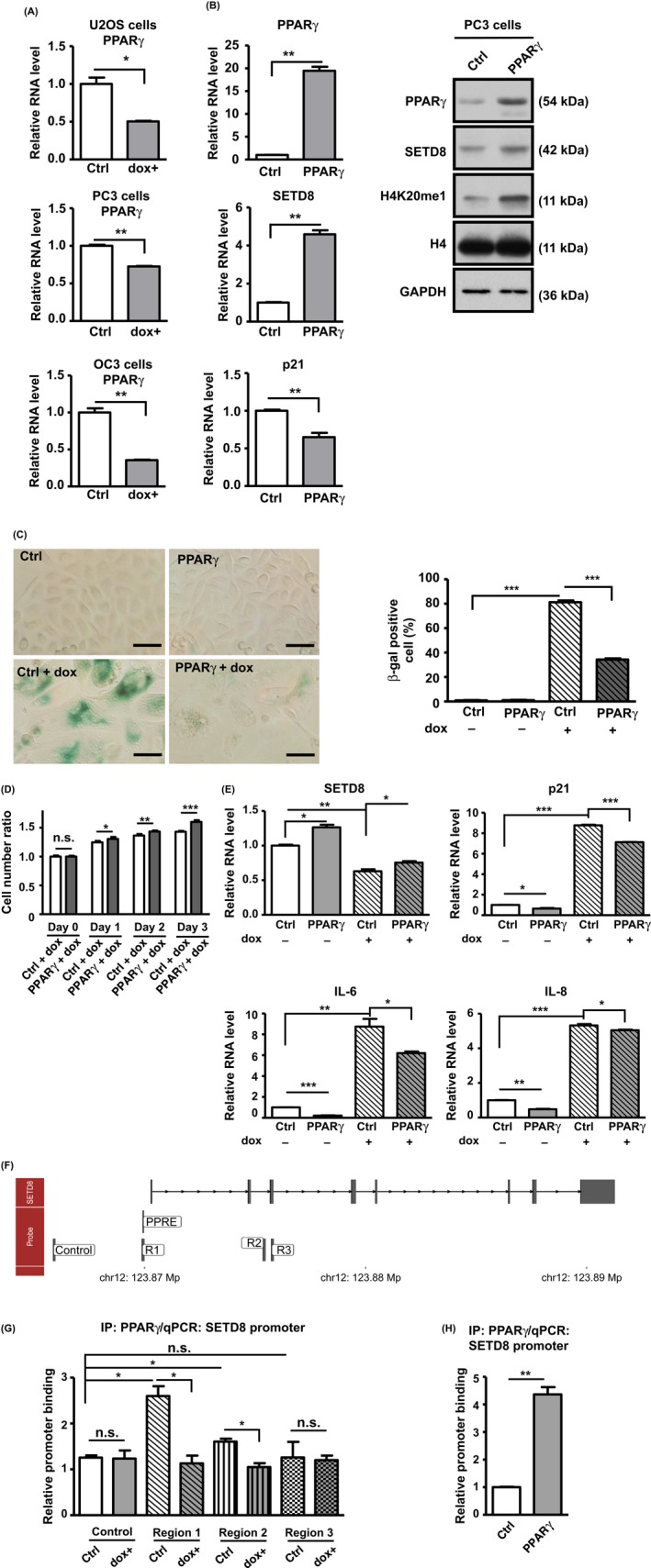
Negative regulation of cellular senescence by a PPARγ‐SETD8 axis. (A) PPARγ mRNA levels in control vs. senescent cells were determined by RT‐qPCR (normalized to *GAPDH*). The comparison was done in U2OS, PC3 and OC3 cells with mock (Ctrl) or dox (dox+) treatment. (B) The PC3 cells were ectopically transfected with empty (Ctrl) or PPARγ cDNA plasmid for 3 days. Expression levels of PPARγ, SETD8 and p21 mRNA were then measured by RT‐qPCR (left). Data were normalized the levels of *GAPDH* and shown relative to the Ctrl group. Immunoblotting was also performed to assess the expression of PPARγ, SETD8 and H4K20me1 in the indicated groups (right). (C) to (E) Effect of PPARγ overexpression in counteracting cellular senescence. Upon transfection with the Ctrl or PPARγ‐expressing plasmids, PC3 cells were treated with dox for up to 6 days to induce cellular senescence. Cells were then subjected to β‐gal staining for senescence (scale bar = 50 μm) (C). Bar graphs below show the percentage of β‐gal+ cells in the indicated culture. (D) Cells were also counted for the first 3 days to show the proliferation rate. Ratios were relative to the day 0 point. (E) Abundance of two SASP markers (IL‐6 and IL‐8), SETD8, and p21 was also determined by RT‐qPCR in the indicated culture. Relative levels are shown in comparison to the mock group. (F) Schematic depiction of the *SETD8* gene locus. Locations of the upstream PPRE site as well as the PCR amplicons designed for the PPARγ ChIP experiments are shown (in the Probe track): control, R1, R2, and R3. (G) ChIP analysis of PPARγ occupancy of *SETD8* promoter on chromatin was performed. Chromatin fragments isolated from PC3 cells, under mock (Ctrl) vs. dox treatment culture (3 days), were immunoprecipitated using control (IgG) or PPARγ antibodies. The precipitated DNA fragments were quantitatively analyzed by real‐time PCR using the primers denoted in (F), and normalized to the values of IgG. Control primer serves as nonbinding control. (H) Upon transfection with the control (Ctrl) or PPARγ‐expressing plasmids, ChIP assay was performed as in (G). Real‐time PCR was performed using the primers denoted in region 1 (F). Values of bound DNA were normalized to IgG and presented relative to Ctrl. For all data shown in this figure, at least three independent experiments were performed, with values representing mean ± SE (n.s.: not significant; *P < 0.05; **P < 0.01; ***P < 0.001).

Based on the previous findings that implicated PPARγ in SETD8 expression (Wakabayashi *et al*., 2009), we next speculated the possible involvement of PPARγ in controlling SETD8 expression and associated function in senescence. To this end, we first monitored the expression patterns of PPARγ over the course of senescence induction and subsequently observed that, in strong correspondence to SETD8, PPARγ mRNA transcripts were downregulated in response to dox treatment (Fig. 6A). In support of its role as an upstream regulator, ectopic overexpression of PPARγ led to an increase in the expression of SETD8 and H4K20me1, and conversely a decline in the *p21* mRNA levels (Fig. 6B). Similar results were also obtained by treating cells with rosiglitazone (ROSI), a PPARγ agonist (Fig. S16A). To further demonstrate the role of PPARγ in controlling senescence, we showed that overexpression of PPARγ alleviated the extent of dox‐induced cellular senescence, in terms of β‐gal‐positive staining (Fig. 6C), cell proliferation arrest (Fig. 6D), and SASP marker induction (Fig. 6E). Consistently, upregulation of PPARγ activity by rosiglitazone treatment also abrogated the dox‐mediated, senescence‐associated β‐gal staining, and expression of the SASP markers (Fig. S16B,C).

To further strengthen the notion that PPARγ is directly involved in *SETD8* regulation during cellular senescence, we next aimed to demonstrate that this transcriptional role is mediated through promoter binding. Through sequence analysis, we first identified conserved PPAR‐binding sequence (PPRE) (Wakabayashi *et al*., 2009) within the *SETD8* promoter (Fig. 6F). This consensus regulatory site resides in a homologous region, to which PPARγ was previously shown to bind in the mouse cells line. We next conducted ChIP assay to confirm PPARγ's promoter binding in a chromatin context. Via real‐time PCR analysis, we detected a specific and significant enrichment in the anti‐PPARγ immunoprecipitates of chromatin fragments corresponding to the PPRE‐containing promoter region (Fig. 6G). As controls, we examined upstream control and intragenic sequences, which conversely were not precipitated by the PPARγ antibodies. Furthermore, this promoter occupancy underwent a decline in cells undergoing senescence (Fig. 6G), parallel to the progressive decrease in the abundance of SETD8 mRNA transcripts. By contrast, via ChIP‐qPCR assay on the PPARγ‐overexpressing cells, we consequently found that the chromatin binding of PPARγ increases on *SETD8* promoter upon ectopic expression (Fig. 6H). Collectively, these observations illustrated that PPARγ exerts a positive regulation toward *SETD8* mRNA expression in proliferating cells and that this PPARγ‐SETD8 regulatory axis impinges on the senescence‐associated *p21* expression.

### SETD8‐mediated regulation of senescence in a noncancer cell context

To further substantiate the physiological relevance of SETD8 in senescence regulation, we next set out to recapitulate our earlier observations in a noncancerous context. For that purpose, we employed a normal fibroblast line IMR90 as an independent and genetically stable cellular model and performed several lines of experiments as above. First, with regard to gene expression in response to senescence induction, we found that SETD8 and H4K20me1 underwent a reduction in IMR90 upon dox treatment (Fig. S17A–D), thereby confirming the observations in cancer cells. Interestingly, we also noted a discernable decrease in SETD8 expression in prolonged culture of IMR90 (late vs. early passage) (Fig. S17E), implying similar regulation in cells undergoing replicative senescence. Conversely, expression of p21 was elevated in both contexts (Fig. S17C,D). Coincidently with this dox‐induced upregulation, the abundance of the repressive H4K20me1 marks at the p21 gene loci declined, as shown by ChIP (Fig. S17F). Collectively, these expression patterns are in line with the observations in cancer cells and supported a general downregulation of SETD8 activity in senescent cells. Second, as in the cancer cells, mere knockdown of SETD8 sufficiently triggered senescence of the IMR90 culture (Fig. S18A,B), as revealed by the upregulation of several cellular hallmarks of senescence (Fig. S18C–E). This effect was dependent on p21, as simultaneous knockdown of p21 stalled the progression of senescence in the absence of SETD8 (Fig. S18). Finally, we also demonstrated an antisenescence function of PPARγ in the IMR90 cells—overexpression of PPARγ in the dox‐treated cells alleviated the progression of cellular senescence and also the upregulation of p21 expression (Fig. S19). Viewed together, our results strengthened the extensive implication of SETD8‐mediated regulation in the senescence pathways.

## Discussion

When proliferative cells, such as those arise in transformed tissues, are exposed to exogenous genome‐insulting stresses or aberrant activation of oncogenic signaling, cellular senescence typically ensues to confer a protective response (Campisi & d'Adda di Fagagna, 2007; Pazolli & Stewart, 2008). While several key aspects of gene regulation have been known to underlie this programmed cessation of cell growth, much less is understood about the involvement of the chromatin‐associated epigenetic modifiers in mediating changes in the senescence state of the cells. Using a systems approach that is based on targeted gene profiling, we uncovered in the current study the histone methyltransferase SETD8 as a target as well as a key regulator of the cellular senescence signaling. More significantly, our findings delineated a new pathway through which the PPAR**γ**‐SETD8 axis directly silences *p21* expression at the chromatin level and consequently impinges on its senescence‐inducing function. In particular, SETD8 mediates its repressive effect via maintaining the H4K20me1 level at the *p21* locus. Our present study has added a new important cellular role to this multifunctional epigenetic writer and may provide mechanistic explanation for SETD8's association with tumorigenesis.

### Epigenetic components of the cellular senescence control

In our targeted approach of identifying epigenetic modulators with altered expression, and presumably functional implications, in the doxorubicin‐induced cellular senescence model, we ultimately detected 17 upregulated and 26 downregulated genes among the 135 preselected chromatin‐related targets (Tables S1 and S2). Several epigenetic modulates previously reported to control senescence progression, such as DNMT1 (Cruickshanks *et al*., 2013), DNMT3B (Dodge *et al*., 2005), EZH2 (Fan *et al*., 2011), and HDAC9 (Mason *et al*., 2004),were uncovered by our systematic search, thus illustrating the feasibility of our experimental paradigm. By adding SETD8 to this expanding group of important senescence effectors, our work further reinforces the emerging role of epigenetic regulatory network in the transition of the proliferative states of the cells. Furthermore, there are additional candidates unveiled by our screen that could potentially contribute to the cell senescence process and thus may be considered as targets of future investigation. For instance, another histone methyltransferase, SETD2, which specifically trimethylates Lys‐36 of histone H3 (H3K36me3), was also reduced in expression upon dox treatment. This enzyme activity has been implicated in the control of homologous recombination and DNA repair (Carvalho *et al*., 2014), therefore underlying genome stability. In addition, given that SETD2 is required for the G2/M transition (Carvalho *et al*., 2014) and that the H3K36me3 mark is associated with lifespan in the yeast model (Sen *et al*., 2015), SETD2 may conceivably be another critical component of the epigenetic regulation of cellular senescence. Viewed together, our targeted screening approach has initiated an important basis for systematically understanding the connection between gene regulation at the chromatin level and cellular response to senescence signaling.

### SETD8 is associated with DNA damage signals‐induced cellular senescence

Cellular senescence may be elicited by multiple factors, such as telomere shortening or dysfunction (d'Adda di Fagagna *et al*., 2003), DNA‐damage signaling, or oncogene activation (Halazonetis *et al*., 2008; Gorgoulis & Halazonetis, 2010). In the present study, we focused on the DNA damage‐induced senescence pathway and used doxorubicin as the inducer (Chang *et al*., 1999). While a link of SETD8 to the dox‐induced senescence was unequivocally demonstrated, the extent of SETD8's involvement in the senescence process (general vs. factor‐specific) remains an unresolved issue. To address this, we subjected cells to two additional genome‐insulting stresses, namely UV (Briganti *et al*., 2014) and H_2_O_2_ (Chien *et al*., 2000) as alternative means to induce senescence (Fig. S20A). Expression profiles of the senescence‐associated markers verified the transition into the senescence state (Fig. S20B). Importantly, we found that the RNA levels of both SETD8 (Fig. S20C) and PPARγ (Fig. S20D) were significantly reduced in either experimental senescence models, correlating with the results of the dox treatment experiments. These observations, together with the findings the mere knockdown of SETD8 expression engenders senescence progression even in the absence of any inducer (Fig. 2, Figs S4 and S18), strongly hinted at a more widespread involvement of SETD8 in this cellular process.

Several observations additionally strengthen the scenario for an extensive role of the PPARγ‐SETD8‐H4K20me pathway in cellular senescence. Consistent with our observations, reduction in PPARγ expression in the presence of multiple DNA damage signals was reported previously (Tachibana *et al*., 2005; Briganti *et al*., 2014), whereas rescuing the activity of PPARγ was found to moderate UV‐induced senescence (Briganti *et al*., 2014). These reports further provide a plausible explanation for the stress‐associated downregulation of PPARγ and consequently the associated downstream network—the oxidative imbalance associated with DNA damage may activate the forkhead box protein O (FoxO1), which is a known transcriptional repressor of PPARγ (Dowell *et al*., 2003; Briganti *et al*., 2014). Interestingly, recent studies have also shown that the H4K20me1 mark may be in association with senescence in both cell (Chicas *et al*., 2012) and animal (Wang *et al*., 2010) models, exhibiting an inverse relationship with multiple factor‐induced senescence (Chicas *et al*., 2012). Further in line with this presumed connection with senescence, public genomewide data also evidenced an enrichment of H4K20me1 in the promoter regions of genes encoding SASP markers (Gorgoulis & Halazonetis, 2010; Nardella *et al*., 2011), such as IL‐6 and TNFRSF1A (Fig. S21A,B). Collectively, these findings lend strong support to the notion that the PPARγ‐SETD8‐H4K20me pathway and its epigenetic effect constitute a key checkpoint mechanism that underlies DNA damage sensing and cellular proliferation control.

### The mode of SETD8‐mediated regulation of *p21*

One of the intriguing findings of our study is that, in spite of the prior lack of evidence linking SETD8 to the H4K20me1 level at *p21* promoter (Shi *et al*., 2007), SETD8 actually exhibits occupancy of the *p21* promoter and controls its epigenetic state (Fig. 5). This discrepancy in findings between the previous and current studies may arise possibly due to distinctions in the experimental design: (i) By cross‐referencing the *p21* promoter region analyzed in the ChIP experiments of the earlier study (Shi *et al*., 2007) with ENCODE/UCSC Genome Browser (http://genome.ucsc.edu/), we found that this amplicon does not coincide with the annotated H4K20me1 marker profile. Our ChIP results instead showed a biased distribution of SETD8 and H4K20me1 in the *p21* gene body, particularly in the PC3 cells (Fig. 5). (ii) The biological contexts in which SETD8 was dissected were different—while the earlier report elucidated functional relevance of the SETD8‐mediated methylation at Lys382 of p53 in apoptosis (Shi *et al*., 2007), our work focused on the connection of SETD8 to *p21* expression during cellular senescence.

Despite this revelation of a direct role of SETD8 in *p21* expression, our present work does not exclude possibility that the SETD8‐mediated control may still engage other regulatory factors (Fig. S22). In this regard, the senescence‐associated *p21* expression is reportedly subject to regulation by the coordinated action of PRC2 complex protein EZH2 and HDAC1 (Fan *et al*., 2011). By virtue of an interaction with another PRC2 component, SUZ12 (Qin *et al*., 2013), SETD8 may presumably cooperate with these epigenetic silencers in exerting the transcription repressive function. In addition, recent work by West *et al*. has implicated the L3MBTL1 protein as a ‘reader’ for both the p53 Lys382 methylation and H4K20me marks and in this capacity serves as a regulator of the downstream *p21* and *PUMA* transcript levels (West *et al*., 2010). Incidentally, both of these modifications are targeted by SETD8, therefore implying that SETD8 may synergize with L3MBTL1 in this functional context. Although our existing results are consistent with a p53‐independent role of SETD8 in senescence (Fig. 3), it remains a formal possibility that, in the presence of wild‐type p53, the combined action of SETD8 and other factors may provide yet additional layer of gene regulation.

### SETD8‐based senescence therapy

Given that senescence is a stress protective response that culminates in a permanent proliferative arrest, targeting or enhancing this process has emerged as a viable tumoricidal treatment option (Collado & Serrano, 2010). Indeed, several pro‐senescence therapies are currently under development or being tested clinically (Nardella *et al*., 2011). As our current work has clearly illustrated an antisenescence function of SETD8, inhibition of this activity may therefore be utilized as part of a senescence‐based treatment regimen. To this end, several SETD8‐targeting microRNAs, such as miR‐502, miR‐7, or miRNA‐127‐3p (Milite *et al*., 2016), have been shown to abrogate malignant attributes of cancer—proliferation, EMT, and invasion. In addition, multiple compounds recently identified to target the activity of SETD8 are proven effective inhibitors *in vitro* (Milite *et al*., 2016). Additional testing of these possibly therapeutic molecules in animal models and clinical setting should reveal their potential in treating tumor and further underscore the central role of SETD8 in cell proliferation control.

## Experimental procedures

### Plasmids and siRNAs for ectopic expression

A plasmid encoding hSETD8 was constructed by first PCR amplification of hSETD8 coding region (BamH1‐SET8F: GGATCCATGGCTAGAGGCAGGAAG. Hindlll‐SET8R: AAGCTTTTAATGCTTCAGCCACG). This cDNA fragment was then subjected to digestion with BamH1 and HindIII, followed by ligation into the BamHI/HindIII sites of the pcDNA 3.1/myc‐His(−) A vector (Invitrogen, Carlsbad, CA, USA). For transient knockdown of target genes, cells were transfected with control or specific siRNAs with lipofectamine RNAiMAX (Invitrogen). The target‐specific siRNAs used are as follows: SETD8 (Ambion, Carlsbad, CA, USA s51988 and s51989); c‐Myc siRNA (Ambion s9129 and s9130); TP53 siRNA (Ambion s605); p21(CDKN1A) siRNA (Ambion s415); and PHF2 siRNA (Invitrogen HSS170929, HSS170930, and HSS170931). Negative control siRNAs were also purchased from the respective manufacturers.

### Cell culture

Human primary normal lung fibroblast IMR90 (ATCC number CCL‐186) was maintained in minimum essential medium (Invitrogen) with 10% FBS. OC3 is an indigenous oral carcinoma cell line established in Taiwan. U2OS cells (osteosarcoma; ATCC number HTB‐96) were cultured in DMEM, supplemented with 10% FBS, 2 mm l‐glutamine, 100 units mL^−1^ penicillin, and 100 μg mL^−1^ streptomycin. PC3 cells (human prostate cancer) were maintained in RPMI, with 10% FBS, 2 mm l‐glutamine, 100 units mL^−1^ penicillin, and 100 μg mL^−1^ streptomycin. For activation of PPARγ, the agonist rosiglitazone (ROSI) was used (100 nm; Sigma, St Louis, MO, USA) (Herroon *et al*., 2013).

### DNA damage‐induced cellular senescence

Several DNA‐damaging agents reportedly may induce cellular senescence—doxorubicin (dox) (Chang *et al*., 1999), UV (Briganti *et al*., 2014), or H_2_O_2_ (Chien *et al*., 2000). Dox‐induced senescence in multiple cell lines was used initially as reference (Chang *et al*., 1999). For dox treatment, differential doses were used depending on the cell lines (50 nm in OC3 cells; 50 nm in U2OS; 200 nm in PC3). UV irradiation of OC3, U2OS, or PC3 cells was performed using the UVB broadband light sources (Vilber Lourmat BLX‐E254), respectively, at the levels of 0.001, 0.001, and 0.002 J cm^−2^. H_2_O_2_ was used, for 2 h, at the following concentrations: OC3 cells, 200 nm; U2OS cells, 200 nm; and PC3 cells, 400 nm. Upon treatment, or at the indicated time points, cells were collected for senescence assessment.

### Establishment of a targeted gene expression‐profiling screen for senescence‐associated epigenes

To identify epigenetic modulators with putative roles in cellular senescence, a total of 135 epigenetic modulators were manually selected for this screen. Using the 200 epigenetic genes reported by Poleshko *et al*. (Poleshko *et al*., 2010) as reference, this target set was further selected based on functional annotation in KEGG BREITE database. Finally, we defined six epigenetic subfamilies according to enzymatic activities: 45 chromatin remodeling factors, nine DNA methylation‐associated factors, 19 histone acetyltransferases, 13 histone deacetylases, 41 genes involved in histone methylation, and eight histone demethylation enzymes. Gene expression profiling was based on RT‐qPCR and performed on RNA samples derived from cells treated with low concentration of doxorubicin, which is a classical model of induced cellular senescence (Chang *et al*., 1999). Relative expression of the selected target epigenes was determined by normalization to three internal controls—*ERGIC1, B2M* (β2 microglobulin), and *EEF1A1*, which all exhibited unfluctuating expression between different treatment groups.

### SA‐β‐Gal staining

Cells were fixed by incubation in 2% formaldehyde/0.2% glutaraldehyde in PBS for 5 minutes at room temperature and stained for SA‐β‐Gal activity in X‐gal solution [1 mg mL^−1^ X‐gal, 0.12 mmol L^−1^ K_3_Fe(CN)_6_, 0.12 mmol L^−1^ K_4_Fe(CN)_6_, 1 mmol L^−1^ MgCl_2_ in PBS at pH 6.0] at 37 °C for 18 h. After 1× PBS washes, microcopy images were then taken.

### RNA isolation and quantitative reverse transcription PCR (RT‐qPCR)

Total RNA from cells was isolated using the TRIzol reagent (Invitrogen) according to manufacturer's instructions. For target mRNA quantification, RNA samples (1 μg) were first converted into corresponding cDNA by RT reaction (MMLV; Invitrogen), in which oligo‐dT primers were used. RT reaction with 10 μL of total mixture will be carried out using the following conditions: 42 °C for 90 min, followed by 70 °C for 15 min and 25 °C 30 min. RT products were diluted 20‐folds for subsequent quantitative PCR analysis. All qPCR reactions were performed in 1 × SYBR Master Mix (Applied Biosystem, Foster City, CA, USA) on ABI Prism 7500 or 7900 Fast Real‐Time PCR System (Foster City, CA, USA). Primers used in real‐time PCR experiments are listed in Table S3. For raw Ct values greater than 35, they will be converted to 35. The raw Ct data were then normalized by global median normalization before further analysis. For relative mRNA expression, the average Ct of internal control (*GAPDH*) was subtracted from the raw Ct value to obtain ‐Ct (dCt), which was further converted to 39‐Ct.

### Flow cytometry‐based analysis of cell cycle and apoptosis

To profile cell cycle progression, we performed propidium iodine (PI) stain after doxorubicin treatments. One million cells were washed in phosphate‐buffered saline and then fixed in 70% ethanol at 4 °C for 45 min. The cells were pelleted and resuspended in 500 μL of PI staining solution composed of 50 μg mL^−1^ PI and 0.1 mg mL^−1^ RNase A on ice for 1 h in the dark. PI‐stained cells were analyzed by flow cytometry using a FACSCAlibur instrument with cellquest analysis software (BD Biosciences, San Jose, CA, USA). The extent of apoptosis was monitored by using the Annexin‐V FLUOS Staining Kit (Roche Applied Science, Indianapolis, IN, USA), according to the manufacturer's instructions. Briefly, cells were collected resuspended in 100 mL of Annexin‐V‐FLUOS labeling solution. After incubation at room temperature for 15 min in the dark, cells were analyzed by the flow cytometry using a FACSCAlibur instrument with cellquest analysis software (BD Biosciences).

### Antibodies and Western blot analysis

The specific antibodies against the followings were used in this study: hSETD8 (ab3798 from Abcam, Cambridge, MA, USA, for ChIP; 06‐1304 from Millipore, Billerica, MA, USA, for immunoblotting), H4K20me1 (ab9051 from Abcam), p53 (sc126 from Santa Cruz Biotechnology, Dallas, TX, USA), p21 (05‐345 from Millipore), GAPDH (AP0063 from Bioworld Technology, St Louis Park, MN, USA), and PPARγ (ab41928 from Abcam, for ChIP; 81B8 from Cell Signaling, Danver, MA, USA, for immunoblotting). Western blot analysis was performed after electrophoretic separation of polypeptides by SDS‐PAGE and transfer to Immobilon‐P/PVDF membrane (Millipore). Blots were probed with the indicated primary and appropriate secondary antibodies. Immunobands were subsequently detected by the enhanced chemiluminescence reaction (ECL) (PerkinElmer; Waltham, MA, USA).

### Chromatin immunoprecipitation

ChIP assay was performed as described previously (Yang *et al*., 2012a). Crosslinked, sonicated chromatin was precleared before being incubated with 2.5 μg of indicated antibodies and rotated at 4 °C overnight. For the recovery of chromatin DNA, the following antibodies were used: anti‐H4K20me1, anti‐SETD8, or anti‐PPARγ. Normal rabbit or mouse IgG (Millipore) was used for the mock immunoprecipitation. After extensive washes, immunocomplexes were treated with proteinase K and de‐crosslinked. Bound DNA in the precipitates, as well as input DNA (1/10 fragmented chromatin), was extracted, purified, and subjected to quantitative real‐time PCR analysis for promoter regions (primer sequences are shown in the Table S3).

### Statistical analysis

Data are presented as mean with error bars indicating the standard error (SE). The Student's *t*‐test or two‐way ANOVA with Bonferroni's correction (post‐tests) was used to determine the statistical significance of quantitative comparisons done in all cell biological, gene expression, and ChIP assays. Degrees of statistical significance (n.s., not significant; *P < 0.05; **P < 0.01; ***P < 0.001) are indicated in the respective figure legends.

## Funding info

No funding information provided.

## Conflict of interest

None declared.

## Author contributions

CT Shih designed and executed experiments, performed data analysis, and prepared manuscript; YF Chang, YT Chen, CP Ma, and CC Yang performed experiments and data analysis; JC Lu and YS Tsai contributed important material, HC Chen contributed to initial study concept; BC Tan contributed to experimental design, data analysis, and manuscript preparation, and obtained funding.

## Supporting information


**Fig. S1** Establishment of a dox‐induced cellular senescence model for targeted gene screen.
**Fig. S2** siRNA‐depletion of PHF2 does not affect features of cellular senescence.
**Fig. S3** Expression alteration of SETD8 and H4K20me1 during dox‐induced cellular senescence.
**Fig. S4** Role of SETD8 in senescence induction and *p21* up‐regulation in the p53‐wt U2OS cells.
**Fig. S5** No alteration of *p16(INK4a/CDKN2A)* mRNA levels during dox‐induced or SETD8‐mediated senescence in PC3 cells.
**Fig. S6** Marginal extent of apoptosis in the SETD8 knockdown cells.
**Fig. S7** Ectopic expression of SETD8 abrogates dox‐induced cellular senescence in U2OS and PC3 cells.
**Fig. S8** Expression of *p53* and *SETD8* in knockdown cells. Related to Fig. 3.
**Fig. S9** Depletion of p21 inhibits dox‐induced cellular senescence.
**Fig. S10** p21 is dispensable to the maintenance of the cellular senescence state.
**Fig. S11** Expression of *p21* and *SETD8* in knockdown cells. Related to Fig.S4 and S12.
**Fig. S12** Knockdown of p21 alleviates the senescent state of the SETD8‐depleted PC3 cells. Related to Fig. 4.
**Fig. S13** SETD8 does not associate with the chromatin region of the *IL‐6* and *IL‐8* gene loci.
**Fig. S14** Characterization of possible role of miRNAs in SETD8 expression regulation.
**Fig. S15** c‐MYC is inconsequential in senescence‐associated SETD8 down‐regulation.
**Fig. S16** Activation of PPARγ by Rosiglitazone (ROSI) reverses dox‐induced senescence.
**Fig. S17** Senescence‐associated expression alteration of SETD8 and H4K20me1 in normal fibroblast cells of IMR90.
**Fig. S18** Knockdown of p21 alleviates the senescent state of the SETD8‐depleted IMR90 cells.
**Fig. S19** Negative regulation of cellular senescence by PPARγ in IMR90 cells.
**Fig. S20** SETD8 down‐regulation in multiple DNA damage factors‐induced cellular senescence.
**Fig. S21** H4K20me1 distribution in SASP gene regions.
**Fig. S22** Schematic model for the functional implication of the PPARγ‐SETD8‐H4K20me1 pathway in cellular senescence.
**Table S1** Up‐regulated epigenes in response to doxorubicin treatment.
**Table S2** Down‐regulated epigenes in response to doxorubicin treatment.
**Table S3** Oligonucleotide primers used for real‐time PCR.
**Data S1** Experimental procedures.
