# Supplementary material for: The PPARγ‐SETD8 axis constitutes an epigenetic, p53‐independent checkpoint on p21‐mediated cellular senescence
Source: Aging Cell. 2017 May 17;16(4):797–813. doi: 10.1111/acel.12607 (PMC5506440; doi:10.1111/acel.12607)
Supplement: Supplementary file 1 — Fig. S1 Establishment of a dox‐induced cellular senescence model for targeted gene screen. Fig. S2 siRNA‐depletion of PHF2 does not affect features of cellular senescence. Fig. S3 Expression alteration of SETD8 and H4K20me1 during dox‐induced cellular senescence. Fig. S4 Role of SETD8 in senescence induction and p21 up‐regulation in the p53‐wt U2OS cells. Fig. S5 No alteration of p16(INK4a/CDKN2A) mRNA levels during dox‐induced or SETD8‐mediated senescence in PC3 cells. Fig. S6 Marginal extent of apoptosis in the SETD8 knockdown cells. Fig. S7 Ectopic expression of SETD8 abrogates dox‐induced cellular senescence in U2OS and PC3 cells. Fig. S8 Expression of p53 and SETD8 in knockdown cells. Related to Fig. 3. Fig. S9 Depletion of p21 inhibits dox‐induced cellular senescence. Fig. S10 p21 is dispensable to the maintenance of the cellular senescence state. Fig. S11 Expression of p21 and SETD8 in knockdown cells. Related to Fig.S4 and S12. Fig. S12 Knockdown of p21 alleviates the senescent state of the SETD8‐depleted PC3 cells. Related to Fig. 4. Fig. S13 SETD8 does not associate with the chromatin region of the IL‐6 and IL‐8 gene loci. Fig. S14 Characterization of possible role of miRNAs in SETD8 expression regulation. Fig. S15 c‐MYC is inconsequential in senescence‐associated SETD8 down‐regulation. Fig. S16 Activation of PPARγ by Rosiglitazone (ROSI) reverses dox‐induced senescence. Fig. S17 Senescence‐associated expression alteration of SETD8 and H4K20me1 in normal fibroblast cells of IMR90. Fig. S18 Knockdown of p21 alleviates the senescent state of the SETD8‐depleted IMR90 cells. Fig. S19 Negative regulation of cellular senescence by PPARγ in IMR90 cells. Fig. S20 SETD8 down‐regulation in multiple DNA damage factors‐induced cellular senescence. Fig. S21 H4K20me1 distribution in SASP gene regions. Fig. S22 Schematic model for the functional implication of the PPARγ‐SETD8‐H4K20me1 pathway in cellular senescence. Table S1 Up‐regulated epigenes in response to doxorubic [file ACEL-16-797-s001.pdf]

SUPPORTING INFORMATION

Supplemental Experimental Procedures

EdU labeling assay

To determine S phase progression, EdU (10 uM) was used to pulse-label the culture for 45 minutes. Subsequent fixation-permeabilization-antibody labeling procedure was performed according to the manufacturer's instructions (Click-iT EdU Flow Cytometry Assay Kits; Invitrogen).

Immunofluorescence

Cells were grown on coverslips for this part of experiments. After treatment, the coverslips were washed briefly in PBS and then fixed for 10 min with 4% paraformaldehyde. Permeabilization was done in PBS supplemented with 0.3% Triton X-100 for 10 min, followed by incubation with primary antibodies in PBS supplemented with 3% BSA for 1 h at room temperature and then with Alexa Fluor 488- or 594-conjugated secondary antibodies (1:1,000; Invitrogen) for 1 h at room temperature. Washes using PBS supplemented with 0.05% Tween 20 were carried out between each step. Finally, coverslips were mounted using mounting medium containing DAPI (Vectashield; Vector Laboratories) followed by confocal microscopy analysis.

miRNA short-hairpin expression constructs

Three human miRNAs, hsa-miR-194-5p, hsa-miR-26a-5p or hsa-miR-29a-3p, were selected and constructed into pcDNA6.2 GW/EmGFP-miR plasmid (Invitrogen). First, dsDNA corresponding to miRNAs were obtained by annealing of the following synthetic oligonucleotide primers: hsa-miR-194-5p-F, TGCTGTGTA ACAGCAACTCCATGTGGAGTTTTGGCCACTGACTGACTCCACATGGTTGCT GTTACA, and hsa-miR-194-5p-R, CCTGTGTAACAGCAACCATGTGGAGTCAGTC AGTGGCCAAAACTCCACATGGAGTTGCTGTTACAC; hsa-miR-26a-5p-F, TGCTGTTCAAGTAATCCAGGATAGGCTGTTTTG GCCACTGACTGACA GCCTATCGGATTACTTGAA, and hsa-miR-26a-5p-R, CCTGTTCAAGTAATCCGATA GGCTGTCAGTCAGTGGCCAAAACAG CCTATCCTGGATTACTTGAACAGCA; hsa-miR-29a-3p-F, TGCTGTAGC ACCATCTGAAATCGGTTAGTTTTGGCCACTGACTGACTAACCGAT CAGATGGTGCTA, and hsa-miR-29a-3p-R, CCTGTAGCACCATCTG ATCGGTTAGTCAGTCAGTGGCCAAAACTAACCGATTTCAGATGGTGCTAC.

The annealed fragments were ligated into pcDNA6.2 GW/EmGFP-miR plasmid.

3' UTR reporter assay

The two reporter plasmids pMIR-SETD8 3UTR 1141-1450bp (encompassing miRNA26a and miRNA194 target sites) and pMIR-SETD8 3UTR 2380-2751bp (with miRNA26a and miRNA29a target sites) were constructed in pMIR REPORT^{TM} luciferase respectively by the following primer pairs: Primer 1 Spe I F, ATTACTAGTCAAAGGACAAA GTGCCCTCA, and Primer 1 Mlu I R, ATTACGCGTGACTCACCTGGCTTCCTAGT; Primer 2 Spe I F, ATTACTAGTTCT CACACACTGTCCCTTCA, and Primer 2 Mlu I R, ATTACGCGTGCCTGGCAATAAA TATTTTAATTTG. Co-transfection of the pRL-TK (Renilla luciferase) plasmid (Promega) serves as an internal control. The expression levels of the two reporter proteins were analyzed by using the Dual-Luciferase Reporter Assay System (Promega).

Supplemental Table S1. Up-regulated epigenes in response to doxorubicin treatment

Gene	0 hr ct	4 hr ct	12 hr ct	24 hr ct	48 hr ct	72hr ct	fold_ change 4 hr	fold_ change 12 hr	fold_ change 24 hr	fold_ change 48 hr	fold_ change 72 hr
HDAC9	30.62	28.98	31.13	28.70	27.79	25.34	3.11	0.70	3.77	7.12	38.82
ATRX	25.91	24.59	24.68	24.16	24.05	23.25	2.50	2.35	3.37	3.62	6.31
CDY1	31.53	31.75	31.70	29.98	29.42	28.99	0.86	0.89	2.94	4.34	5.83
JMJD2C	24.53	20.88	22.30	22.54	22.70	22.34	12.49	4.67	3.97	3.54	4.56
CDY2A	33.85	34.78	34.74	32.74	31.68	31.84	0.53	0.54	2.16	4.48	4.03
SETDB1	27.18	26.48	25.97	26.14	25.75	25.46	1.62	2.31	2.06	2.69	3.30
JMJD2B	22.23	20.57	21.65	21.60	21.66	20.51	3.16	1.49	1.55	1.48	3.29
JMJD2A	21.69	20.77	20.83	20.73	21.06	20.10	1.90	1.82	1.94	1.55	3.02
JARID1B	21.62	20.87	20.98	20.72	21.24	20.06	1.68	1.56	1.86	1.30	2.94
JMJD1A	24.17	22.99	23.27	23.11	22.80	22.74	2.25	1.86	2.08	2.58	2.68
SMARCAD1	22.70	21.87	21.77	21.78	21.07	21.43	1.77	1.91	1.90	3.09	2.41
PHC3	22.76	21.12	22.13	22.07	22.37	21.51	3.11	1.55	1.61	1.32	2.39
HDAC5	23.72	22.78	22.85	21.84	22.89	22.54	1.92	1.82	3.68	1.77	2.26
EZH1	24.66	24.50	24.23	24.60	24.34	23.50	1.11	1.34	1.04	1.24	2.23
HDAC6	23.18	21.39	21.45	21.61	21.84	22.04	3.48	3.33	2.99	2.54	2.20
CHD2	21.79	21.48	21.84	21.65	21.08	20.75	1.23	0.96	1.10	1.63	2.06
HDAC3	20.60	20.77	19.91	20.13	19.45	19.59	0.88	1.61	1.38	2.22	2.01

Supplemental Table S2. Down-regulated epigenes in response to doxorubicin treatment

Gene	0 hr ct	4 hr ct	12 hr ct	24 hr ct	48 hr ct	72hr ct	fold_ change 4 hr	fold_ change 12 hr	fold_ change 24 hr	fold_ change 48 hr	fold_ change 72 hr
DNMT1	18.78	19.56	20.37	21.58	21.98	21.51	0.58	0.33	0.14	0.11	0.15
WHSC1	19.42	18.76	19.90	20.69	21.95	21.97	1.58	0.72	0.42	0.17	0.17
MYST3	21.47	22.18	23.06	23.28	23.85	23.61	0.61	0.33	0.29	0.19	0.23
MBD2	18.22	18.66	19.00	19.18	19.20	20.23	0.74	0.59	0.51	0.51	0.25
EP400	25.07	26.98	28.70	29.57	30.27	27.04	0.27	0.08	0.04	0.03	0.26
KAT5	20.72	21.60	21.93	22.46	22.57	22.61	0.54	0.43	0.30	0.28	0.27
CBX2	25.79	26.36	27.24	29.19	28.54	27.64	0.67	0.37	0.09	0.15	0.28
SETD8	21.63	20.89	22.22	23.05	22.99	23.42	1.66	0.67	0.37	0.39	0.29
BAZ1A	21.65	22.06	22.70	22.04	21.93	23.41	0.75	0.48	0.76	0.82	0.30
MBD3	22.16	22.65	24.96	25.67	25.62	23.88	0.71	0.14	0.09	0.09	0.30
SETD2	21.78	22.48	23.10	22.97	22.87	23.43	0.62	0.40	0.44	0.47	0.32
DNMT3B	24.00	23.25	24.01	24.74	25.44	25.52	1.68	0.99	0.60	0.37	0.35
DOT1L	23.92	24.09	26.04	27.23	26.81	25.38	0.89	0.23	0.10	0.14	0.37
ACTL6A	19.02	18.24	19.31	19.59	19.56	20.46	1.71	0.82	0.67	0.69	0.37
PRMT7	20.60	21.10	21.46	21.79	21.59	21.92	0.71	0.55	0.44	0.51	0.40
MLL	20.54	21.63	22.35	23.11	23.19	21.85	0.47	0.28	0.17	0.16	0.40
PHC1	24.95	25.48	26.35	26.82	26.65	26.25	0.69	0.38	0.27	0.31	0.41
SMARCA1	20.29	21.03	21.14	21.18	21.40	21.58	0.60	0.55	0.54	0.46	0.41
BRPF1	21.73	22.03	22.81	23.26	23.26	22.93	0.81	0.47	0.35	0.35	0.43
DNMT3A	23.44	24.09	24.57	24.88	24.78	24.63	0.63	0.45	0.37	0.39	0.44
SETD1A	23.39	24.80	26.28	26.54	25.27	24.58	0.38	0.14	0.11	0.27	0.44
SMARCD1	19.35	19.85	20.46	20.80	20.60	20.50	0.71	0.47	0.37	0.42	0.45
MBD6	24.36	26.07	27.33	28.06	27.91	25.48	0.31	0.13	0.08	0.09	0.46
SUV39H1	22.78	21.67	22.54	23.38	23.41	23.89	2.15	1.18	0.66	0.64	0.46
SCMH1	23.11	23.03	23.98	24.42	24.16	24.17	1.06	0.55	0.40	0.48	0.48
EZH2	23.19	26.53	22.12	22.72	23.56	24.22	0.10	2.10	1.38	0.77	0.49

Supplemental Table S3. Oligonucleotide primers used for real-time PCR.

Name		Sequences 5' to 3'
TP53	Forward	CGAGCACTGCCCAACAA
	Reverse	GAGTTCCAAGGCCTCATT
p21(CDKN1A)	Forward	ACCTTCCAGCTCCTGTAACATA
	Reverse	ATTGAGCACCTGCTGTATATT
IL-6	Forward	TTCGGTACATCCTCGACG
	Reverse	CAGGCAAGTCTCCTCATT
IL-8	Forward	GGGCCAAGAGAATATCCGA
	Reverse	CTAGGGTTGCCAGATTTAACAG
MDM2	Forward	CAACCAATTCAAATGATTGTGCT
	Reverse	GAAGAAATCTATGTGAATTGAGGC
HDAC9	Forward	GGCTCTAGAAGGAGGACA
	Reverse	GTGGAGAATATCTTCTGCAAGT
DNMT1	Forward	AACCGCTGTATCTAGCAAG
	Reverse	CCTCACATTCATCCACCAA
SETD8	Forward	TCTTGTGATTCCACCAATGC
	Reverse	GGACAGGGTAGAAATCCGT
PPARγ	Forward	GCCAAGCTGCTCCAGAAAAT
	Reverse	TGATCACCTGCAGTAGCTGCA
IL-1ß	Forward	TCTCCGACCACCACTAC
	Reverse	AGCCTCGTTATCCCAT
HMOX-1	Forward	CTCAAACCTCCAAAAGCC
	Reverse	TCAAAAACCACCCCAACCC
WSHC1	Forward	AAAGCAACAAGCATGACTC
	Reverse	CTGGTTCTTCCCTTGGTTT
SUV39H1	Forward	TGGAGAAGATTCGCAAGAACA
	Reverse	CACGTAGTCCAGGTCAAAG
SUV39H2	Forward	CATGGAATATGTTGGAGAGGTA
	Reverse	ATGAGACACATTGCCGT
SUV4-20H1	Forward	TTCTTGTTATTATGGAGATGGGTT
	Reverse	CTCTGAGTCCATATTTGCTATTG
SUV4-20H2	Forward	AGGTGACATGCTTCTACG
	Reverse	TCACGCAGCTGGTACTT
PHF2	Forward	TGACCAAGATGGAGCCG
	Reverse	AGGTTTGCTGTTCTTGTACT
PHF8	Forward	CTTGAATGACTCAGATGACGAC
	Reverse	CTTTGCTATCTTGGTTCGC
EEF1A1	Forward	CAATGTGGGCTTCAATGTCAA
	Reverse	CATAGCCGGCGCTTATTTG
ERGIC1	Forward	CTACGACCTCAGCCCCATCA
	Reverse	GGTGAAGGTCCCGCCAAT
B2M	Forward	CTGGAGGCTATCCAGCGTA
	Reverse	TCTCTCCATTCTTCAGTAAGTCAAC
GAPDH	Forward	AAATCCCATCACCATCTTCC
	Reverse	GCAGAGATGATGACCCTTT
CDKN1A region c	Forward	GGTCTGCTGGAACTCTTAG
	Reverse	TAAGCCACCATGTAAGAAGTTT
CDKN1A region 1	Forward	CCCAGTCTCTTTCTGAGAAAT
	Reverse	CCTGAAGAAGGAGGATGTG
CDKN1A region 2	Forward	AGGGAACGGACTGTATGAG
	Reverse	GCTTCCTTGGGAACAAACT
CDKN1A region 3	Forward	ATTAGGAAACTGCTGGACT
	Reverse	CTGTTTGCCACCAGGTAT
SETD8 region c	Forward	GGAAATTCATTAAACGGGTGAC
	Reverse	CAGCTTTGGAGGTGGTAG
SETD8 region 1	Forward	CTAAACTGCTGGGAGACTCA
	Reverse	GACCGGATCTGGGACTT
SETD8 region 2	Forward	GCGTGTTTCTGTGCTGA
	Reverse	TATATAGGAAGAAGACAGCCTACC
SETD8 region 3	Forward	CCCTTCAGGAAGAGAACTCA
	Reverse	GATCAGAACGAGGCCATTT
CDKN2A	Forward	CTCGGGAAACTTAGATCATCAG
	Reverse	TAAATGGACATTTACGGTAGTGGG
IL-6 ch-ip region	Forward	CTTCTTCATAATCCCAGGCTT
	Reverse	ATTTAGCGTTCCAGTTAATTTGTAT
IL-8 ch-ip region	Forward	AATTCACCAAATTGTGGAGC
	Reverse	TTTGTTCCTTATCAAATACGGAGTATG

Supplemental Figure Legends

Fig. S1

(A)

(B)

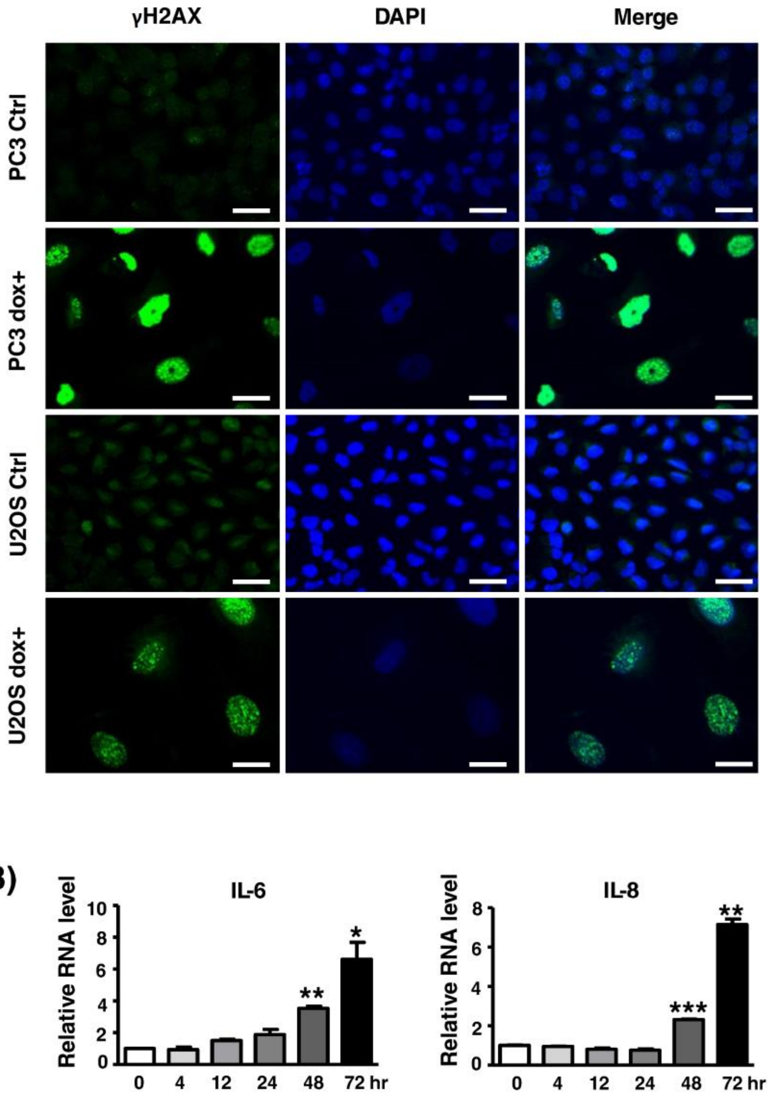

Fig. S1

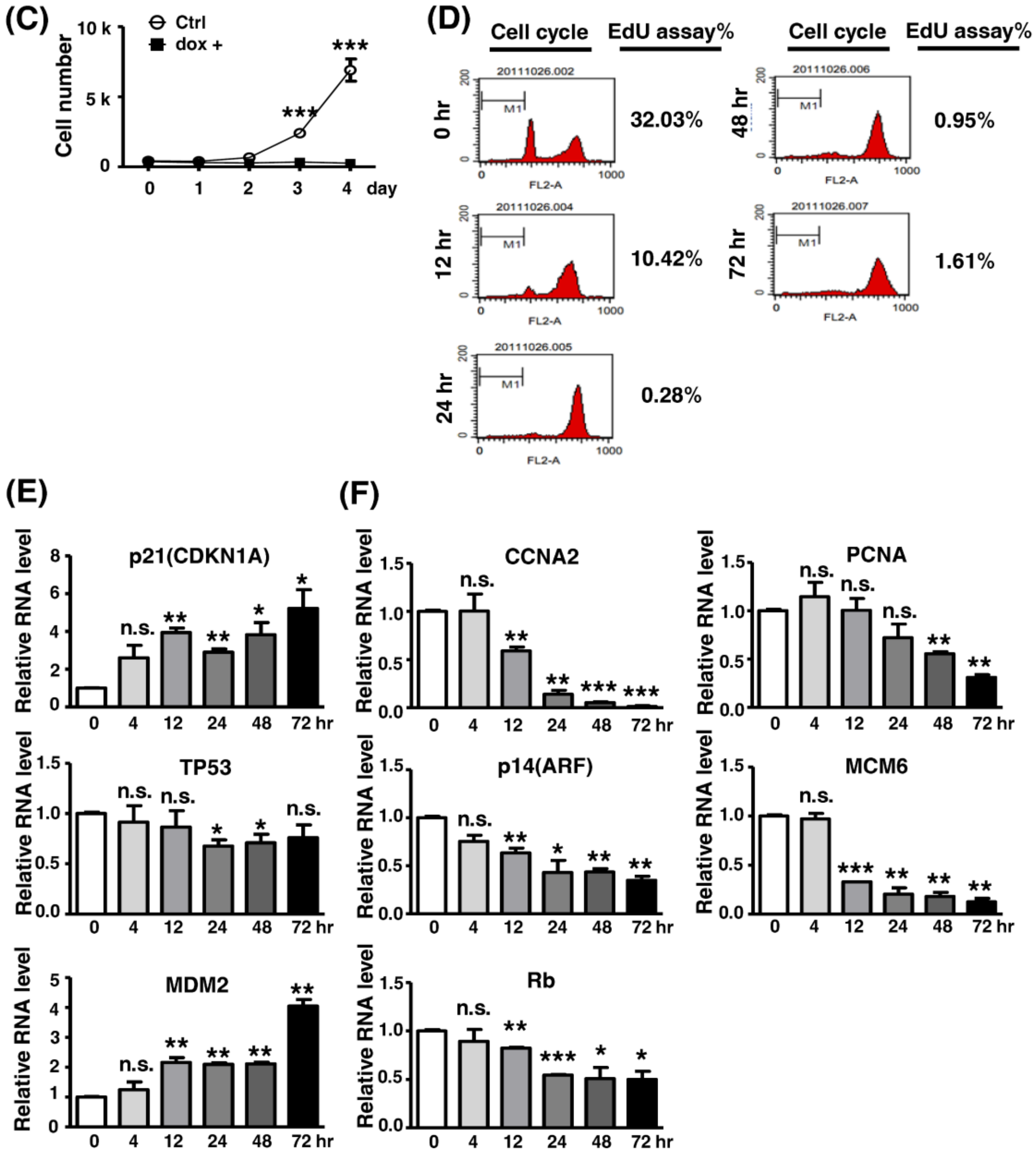

(C)
Ctrl
dox +
Cell number
10 k
5 k
0
0 1 2 3 4 day
(D)
Cell cycle
EdU assay%
0 hr
32.03%
12 hr
10.42%
24 hr
0.28%
48 hr
0.95%
72 hr
1.61%
(E)
(F)
p21(CDKN1A)
CCNA2
PCNA
TP53
p14(ARF)
MCM6
MDM2
Rb
Relative RNA level
0 4 12 24 48 72 hr

Figure S1. Establishment of a dox-induced cellular senescence model for targeted gene screen.

(A) Up-regulation of γH2AX during dox-induced cellular senescence. Upon senescence induction by 7-day dox treatment, PC3 and U2OS cells were analyzed by immunofluorescence assay to illustrate the expression and localization of γH2AX (scale bar, 50 μm). Nuclei were counter-stained by DAPI (shown in blue). **(B)** to **(E)** Senescence induction by low concentration of dox. The OC3 cells were treated with 50 nM of dox for 3 days and subsequently examined for various markers of cellular senescence (see Methods): up-regulation of senescence-associated secretory phenotype (SASP) makers IL-6 and IL-8 as revealed by RT-qPCR **(B)**, cell proliferation curve based on DAPI stained-cell counting **(C)**, cell cycle progression according to flow cytometric analysis (left) or EdU marking of S phase (right) **(D)**, RT-qPCR-based profiling of the mRNA expression of p53 **(E)** or RB-E2F **(F)** downstream targets, as indicated. For **(B)**, **(E)**, and **(F)**, data are normalized to the reference time point (0). All quantitative results shown in Figure S1 are presented as mean ± SE of at last three independent experiments (n.s.: not significant; $*p < 0.05$; $**p < 0.01$; $***p < 0.001$).

Fig. S2

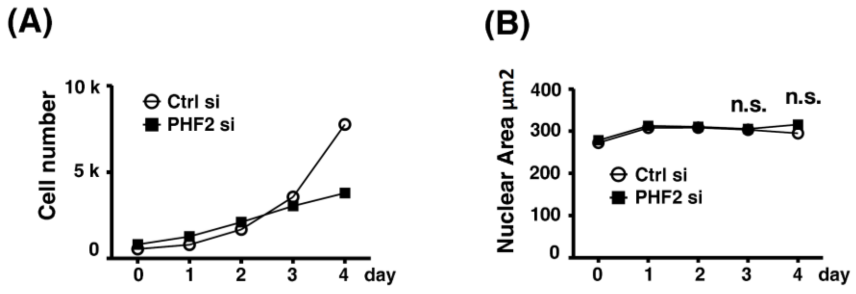

Figure S2. siRNA-depletion of PHF2 does not affect features of cellular senescence.

The U2OS cells were subjected to siRNA-mediated knockdown and analyzed for proliferation rate **(A)** and nuclear size changes **(B)**. Values represent mean ± SE of at last three independent experiments. Ctrl siRNA serves as control. n.s.: not significant.

Fig. S3

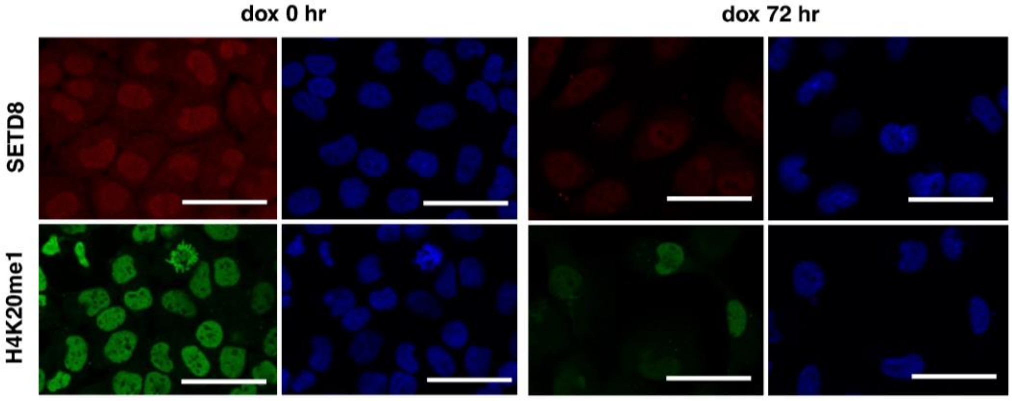

Figure S3. Expression alteration of SETD8 and H4K20me1 during dox-induced cellular senescence.

Upon senescence induction by 3-day treatment of 50 nM doxorubicin, OC3 cells were analyzed by immunofluorescence assay to illustrate the expression and localization of SETD8 and H4K20me1, using the respective antibodies (scale bar, 50 μm). Nuclei were counter-stained by DAPI (shown in blue).

Fig. S4

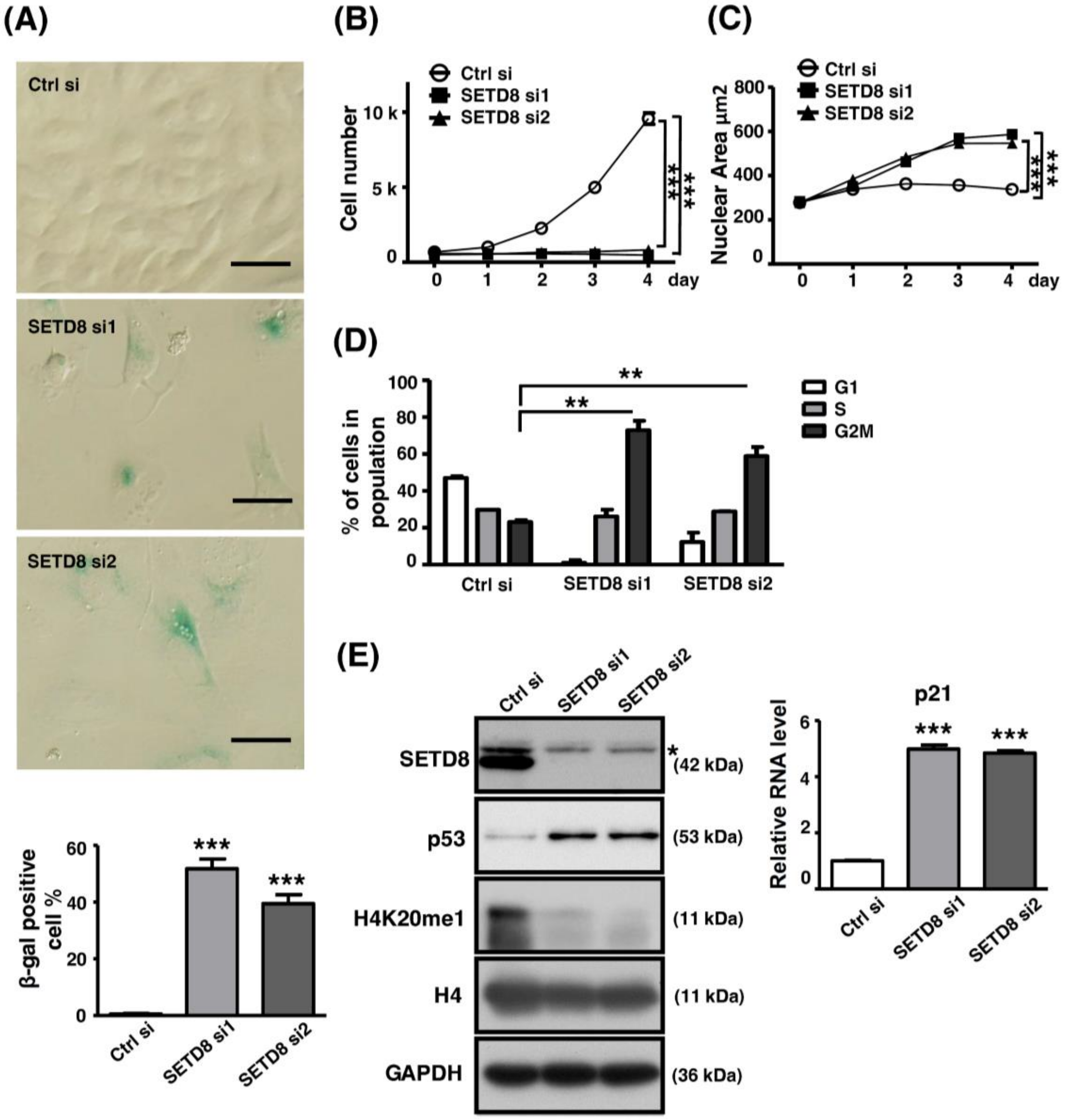

Figure S4. Role of SETD8 in senescence induction and *p21* up-regulation in the p53-wt U2OS cells.

(A) to (E) U2OS cells were transfected with control (Ctrl) or SETD8-targeting siRNAs (si1 & si2), and then subjected to senescence-related phenotype analyses (see Methods): β-gal staining (scale bar = 50 µm for the images, with bar graph below depicting quantitative results) **(A)**, cell proliferation curve **(B)**, nuclear area determination **(C)**, flow cytometry-based cell cycle profiling **(D)**, and *p21* transcript abundance **(E)**. For **(E)**, expression of SETD8 and H4K20me1 were verified by immunoblotting, whereas *p21* levels were determined by RT-qPCR. The asterisk denotes a non-specific signal in the anti-SETD8 blot. All quantitative results shown are presented as mean ± SE of at last three independent experiments (**$p < 0.01$; ***$p < 0.001$).

Fig. S5

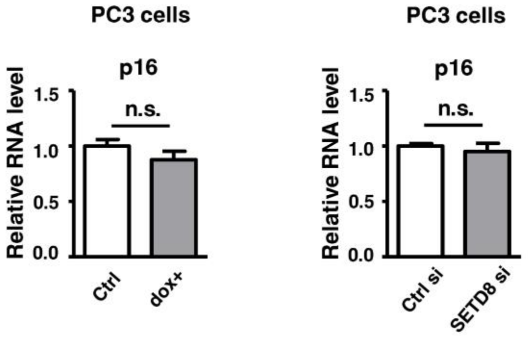

Figure S5. No alteration of *p16(INK4a/CDKN2A)* mRNA levels during dox-induced or SETD8-mediated senescence in PC3 cells.

Senescence was induced in PC3 cells by low concentration of dox or SETD8 depletion, and subsequently monitored by RT-qPCR for the mRNA expression of *p16*. All quantitative results are presented as mean ± SE of at least three independent experiments (n.s.: not significant).

Fig. S6

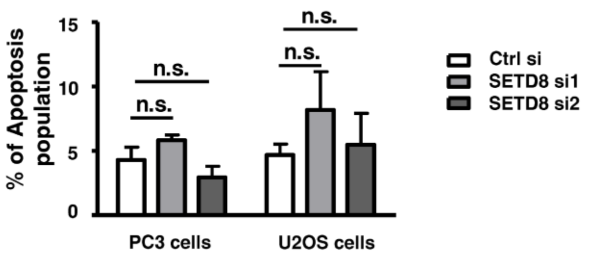

Figure S6. Marginal extent of apoptosis in the SETD8 knockdown cells.
Both U2OS and PC3 cells were transfected with control (Ctrl) or SETD8-targeting siRNAs. After 3-day culture, cells were subjected to Annexin V and PI stain and monitored for apoptosis by flow cytometry. The percentage of apoptotic cells in each group, as indicated, was determined based on the proportion of double-positive cells. Results are presented as mean ± SD of three independent experiments (n.s.: not significant).

Fig. S7

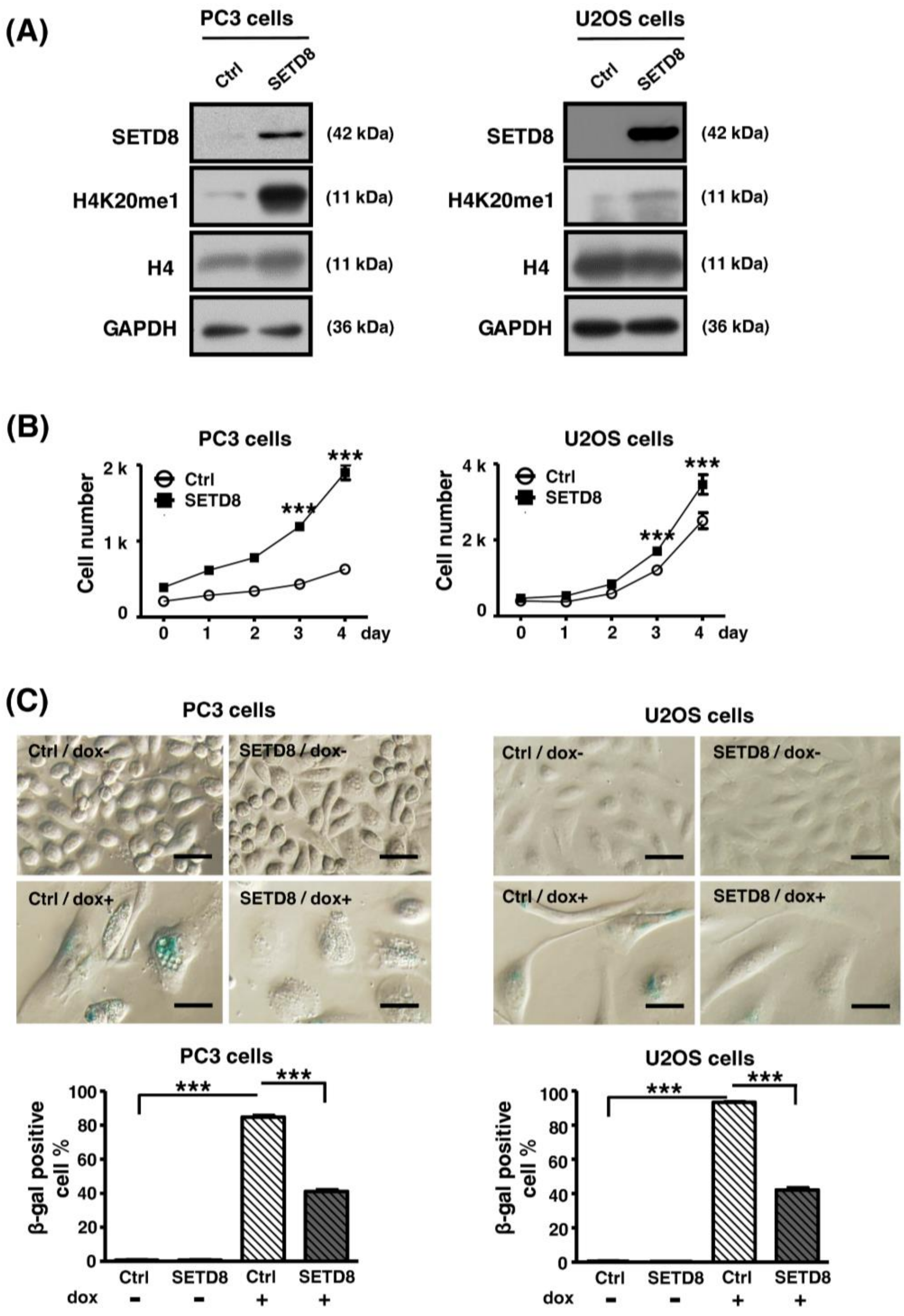
(A)
PC3 cells
Ctrl
SETD8
SETD8
H4K20me1
H4
GAPDH
(42 kDa)
(11 kDa)
(11 kDa)
(36 kDa)
U2OS cells
Ctrl
SETD8
(B)
PC3 cells
Cell number
Ctrl
SETD8
***
2 k
1 k
0
0 1 2 3 4 day
U2OS cells
4 k
2 k
(C)
PC3 cells
Ctrl / dox-
SETD8 / dox-
Ctrl / dox+
SETD8 / dox+
U2OS cells
β-gal positive cell %
100
80
60
40
20
0
Ctrl
SETD8
dox
-
+

Fig. S7

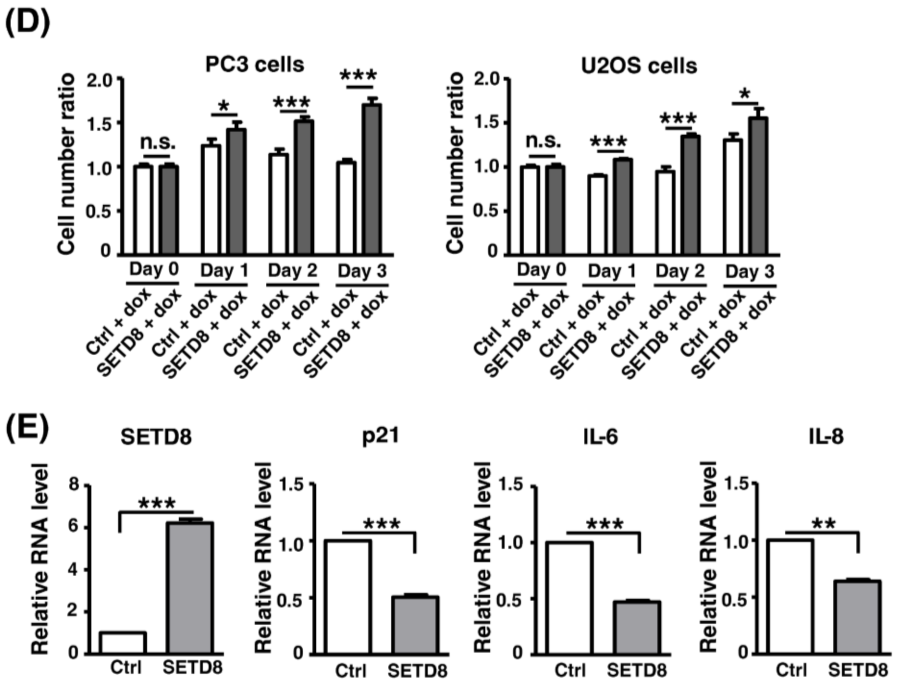

Figure S7. Ectopic expression of SETD8 abrogates dox-induced cellular senescence in U2OS and PC3 cells.

(A) The U2OS or PC3 cells ectopically harboring control or SETD8-expressing plasmid were examined by immunoblotting analysis for SETD8 and H4K20me1 protein levels. GAPDH and histone H4 (H4) serve as internal control. **(B)** Proliferation curve of the cells in **(A)** was determined by a DAPI staining-based counting. Results were normalized to the control group. **(C)** to **(E)** Upon transfection with the SETD8-expressing plasmid as above, U2OS or PC3 cells were treated with dox for 6 days, and subsequently characterized for the extent of senescence induction. This was done according to β-gal stain positivity in **(C)** (scale bar = 50 μm in the microscopy images, with quantitative results shown in the bar graphs), cell proliferation rate **(D)**, and expression of SETD8 and senescence-associated marker genes, as indicated (E). For **(C)**, **(D)**, and **(E)**, data are normalized to the control vector (Ctrl) group. All quantitative results are from at last three independent experiments and presented as mean ± SE of (n.s.: not significant; $*p < 0.05$; $**p < 0.01$; $***p < 0.001$).

Fig. S8

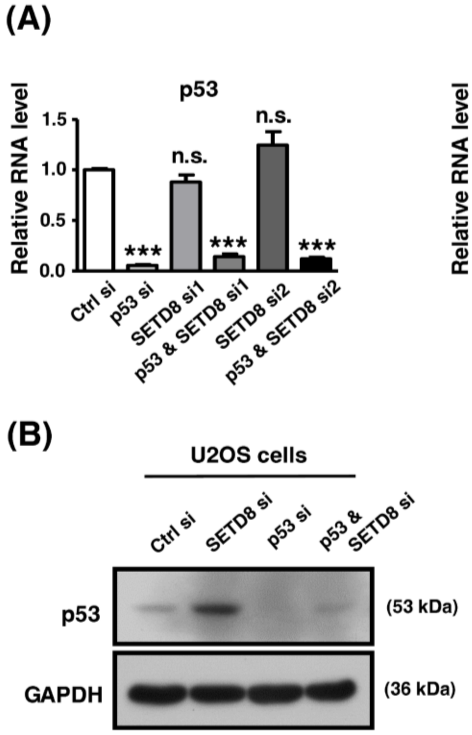

Figure S8. Expression of *p53* and *SETD8* in knockdown cells. Related to Figure 3.

(A) Extent of *p53* (left) or *SETD8* (right) knockdown in cells shown in Figure 3, based on mRNA expression levels, was validated by real-time RT-PCR. **(B)** p53 protein levels in the knockdown cells were assessed by immunoblotting, with GAPDH as the internal control.

Fig. S9

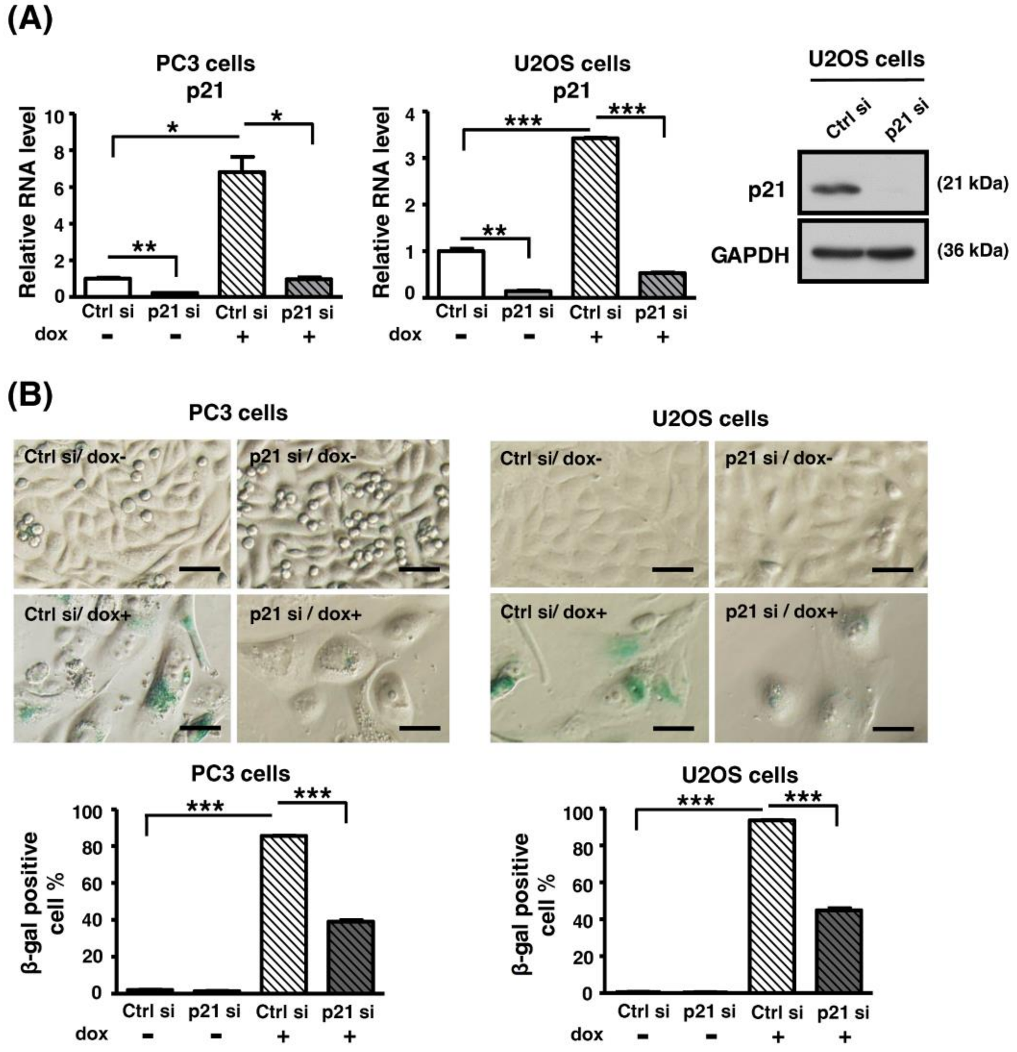

Figure S9. Depletion of p21 inhibits dox-induced cellular senescence.

(A) Expression of *p21* was analyzed by real-time RT-PCR analysis or immunoblotting (rightmost panel) to illustrate knockdown efficiency. **(B)** Extent of senescence induction was examined as above, on the basis of β-gal staining (scale bar = 50 μm). All results are shown as mean ± SE of at last three independent experiments (*$p < 0.05$; **$p < 0.01$; ***$p < 0.001$).

Fig. S10

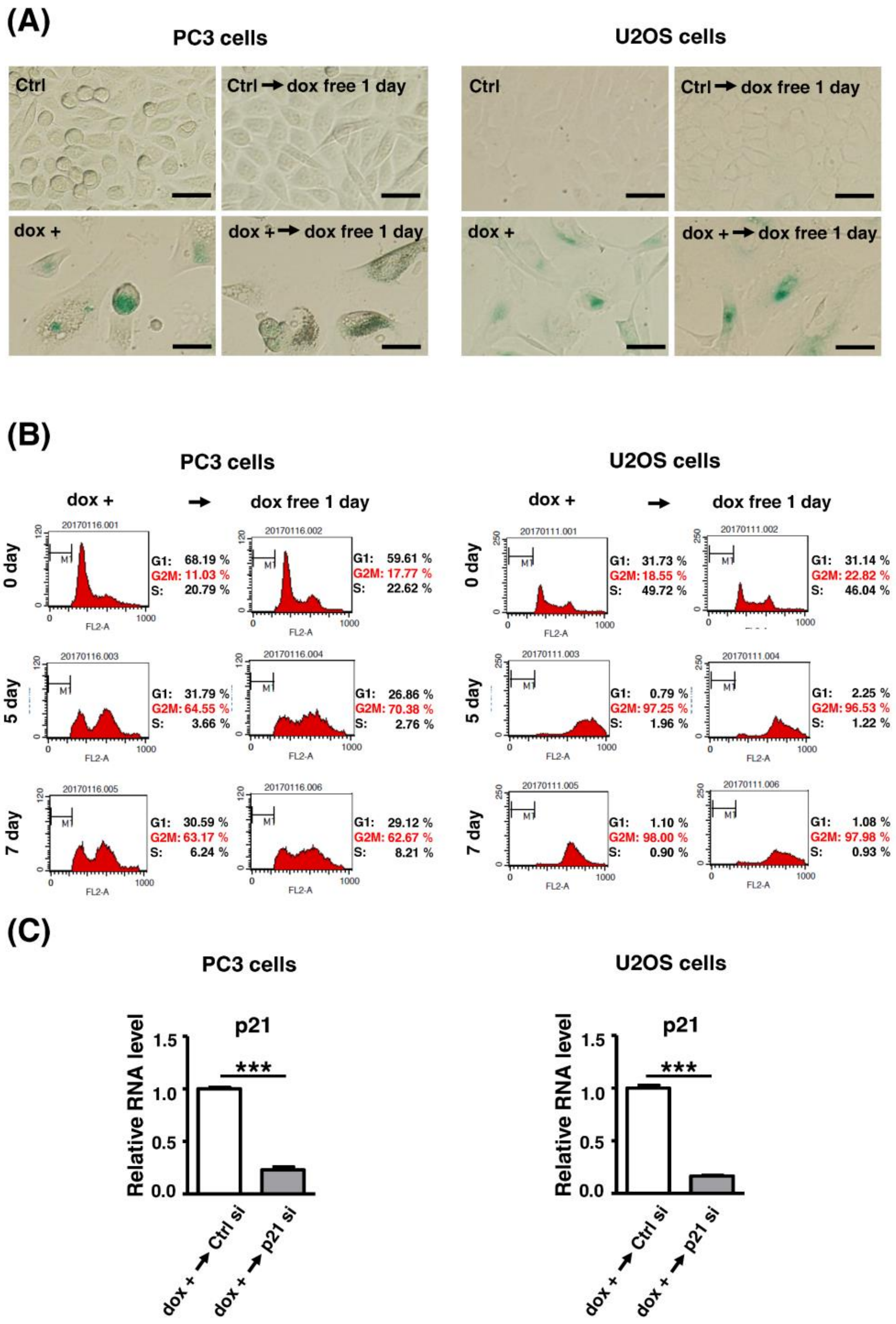
(A)
PC3 cells
U2OS cells
Ctrl
Ctrl → dox free 1 day
dox +
dox + → dox free 1 day
(B)
PC3 cells
U2OS cells
dox +
dox free 1 day
0 day
5 day
7 day
G1: 68.19 %
G2M: 11.03 %
S: 20.79 %
G1: 59.61 %
G2M: 17.77 %
S: 22.62 %
G1: 31.73 %
G2M: 18.55 %
S: 49.72 %
G1: 31.14 %
G2M: 22.82 %
S: 46.04 %
G1: 31.79 %
G2M: 64.55 %
S: 3.66 %
G1: 26.86 %
G2M: 70.38 %
S: 2.76 %
G1: 0.79 %
G2M: 97.25 %
S: 1.96 %
G1: 2.25 %
G2M: 96.53 %
S: 1.22 %
G1: 30.59 %
G2M: 63.17 %
S: 6.24 %
G1: 29.12 %
G2M: 62.67 %
S: 8.21 %
G1: 1.10 %
G2M: 98.00 %
S: 0.90 %
G1: 1.08 %
G2M: 97.98 %
S: 0.93 %
FL2-A
(C)
PC3 cells
U2OS cells
p21
Relative RNA level
***
dox + → Ctrl si
dox + → p21 si

Fig. S10

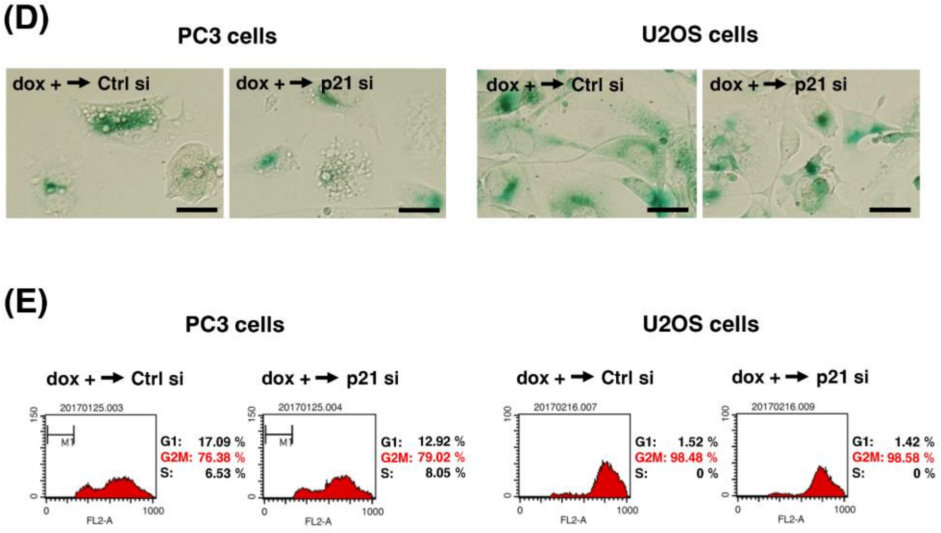

Figure S10. p21 is dispensable to the maintenance of the cellular senescence state. **(A) & (B)** The PC3 and U2OS cells were first treated without (Ctrl) or with dox for 5 or 7 days. To examine the stability of the senescence state, dox was removed from culture media subsequently to the treatment course (denoted as "→ dox free 1 day"). Cells were harvested one day after drug removal and analyzed for β-gal staining **(A)** and cell cycle distribution **(B)**. **(C)** to **(E)** Similar senescence induction experiments (7 days) were done as above, except that after drug removal p21 was depleted by siRNAs **(C)**. Cells were then examined for β-gal staining **(D)** and cell cycle distribution **(E).** RT-qPCR data shown in **(C)** are normalized to the control (Ctrl si) group and quantitatively presented as mean ± SE of at least three independent experiments (***$p < 0.001$).

Fig. S11

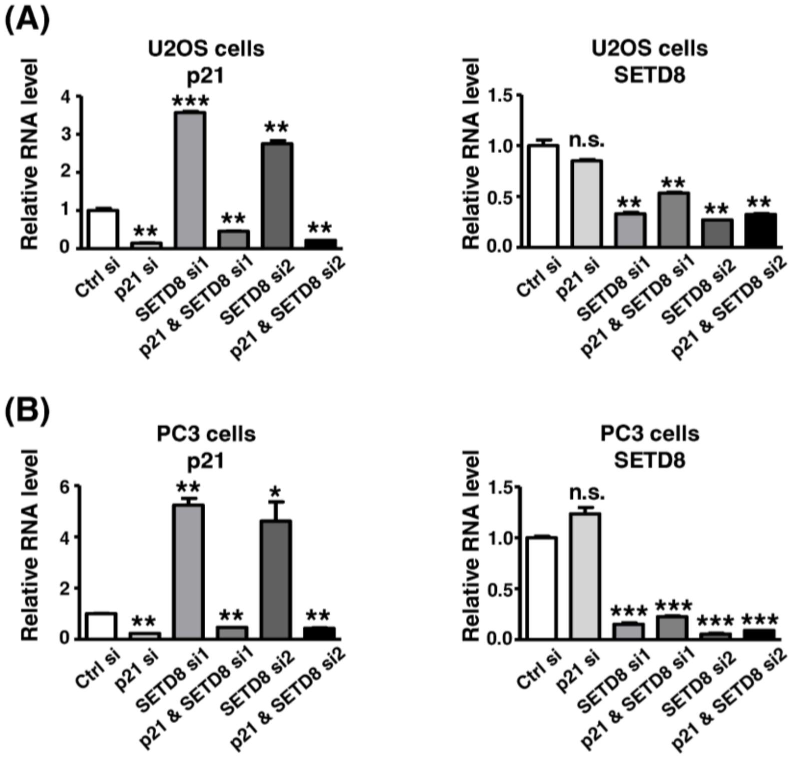

Figure S11. Expression of *p21* and *SETD8* in knockdown cells. Related to Figures 4 and S12.

Extent of *p21* or *SETD8* knockdown in cells shown in Figure 4 **(A)** and Figure S12 **(B)** was validated by real-time RT-PCR. Normalized expression levels of *p21* (left) and *SETD8* (right) are shown (GAPDH as the internal control).

Fig. S12

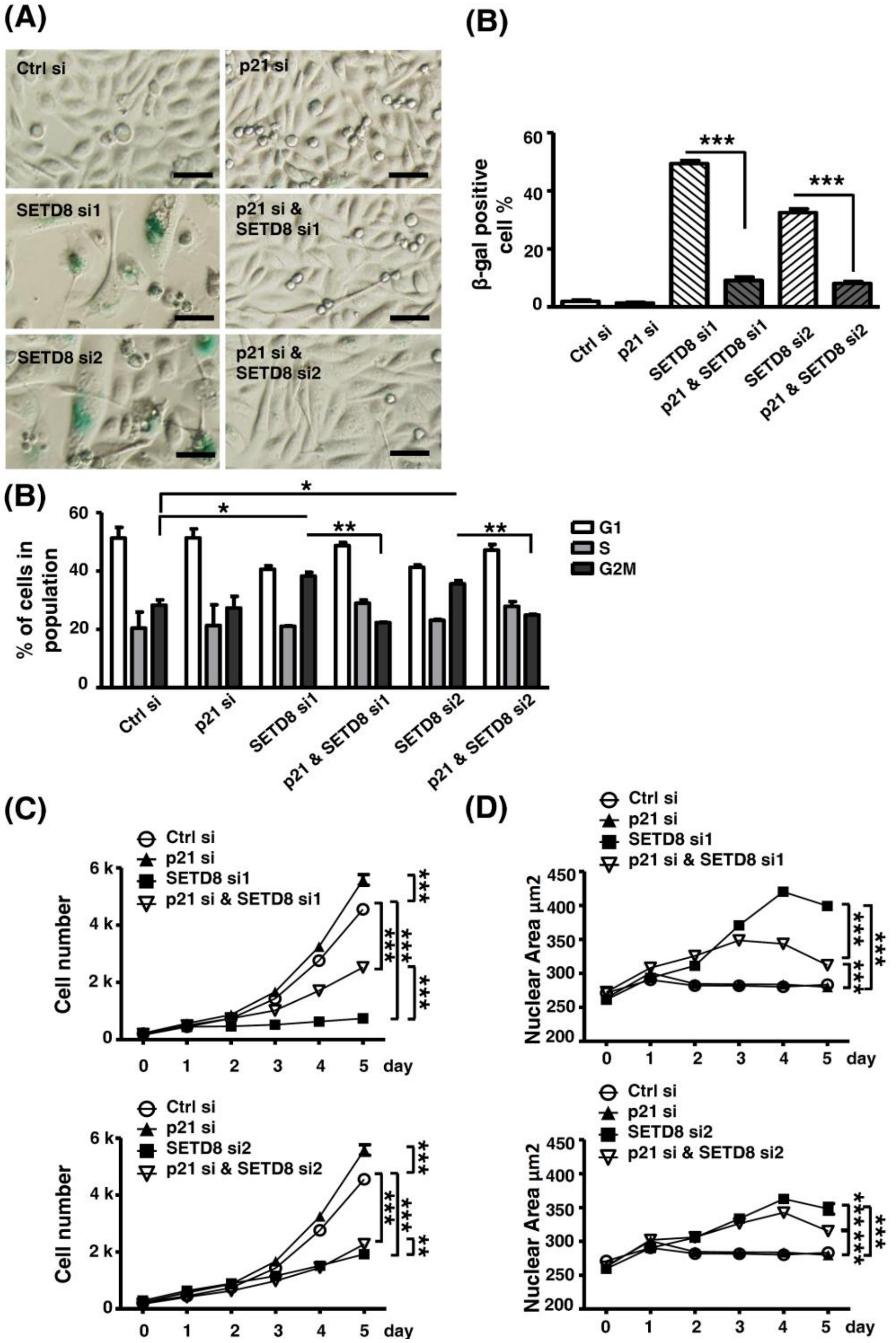

Figure S12. Knockdown of p21 alleviates the senescent state of the SETD8-depleted PC3 cells. Related to Figure 4.

(A) to **(D)** Similar co-knockdown experiments were done as in Figure 4, except using the PC3 line (p53-null). Senescence phenotypes were assessed accordingly: β-gal staining **(A)**, cell cycle distribution **(B)**, cell proliferation **(C)**, and nuclei area **(D)**. All results are shown as mean ± SE of at last three independent experiments (*$p < 0.05$; **$p < 0.01$; ***$p < 0.001$).

Fig. S13

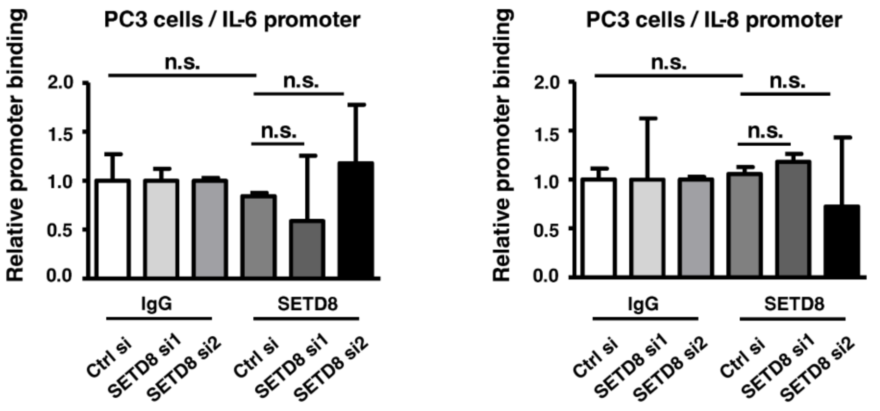

Figure S13. SETD8 does not associate with the chromatin region of the *IL-6* and *IL-8* gene loci.

PC3 cells were transfected with control (Ctrl) or SETD8 siRNAs for 3 days. ChIP analysis was done on chromatin isolated from these cells, using control (IgG), or SETD8 antibodies. The precipitated DNA fragments were quantitatively analyzed by real-time PCR using primer pairs corresponding to the *IL-6* (left) or *IL-8* (right) promoter, and normalized to the values of IgG. Statistical significance of the indicated comparisons: n.s.: not significant.

Fig. S14

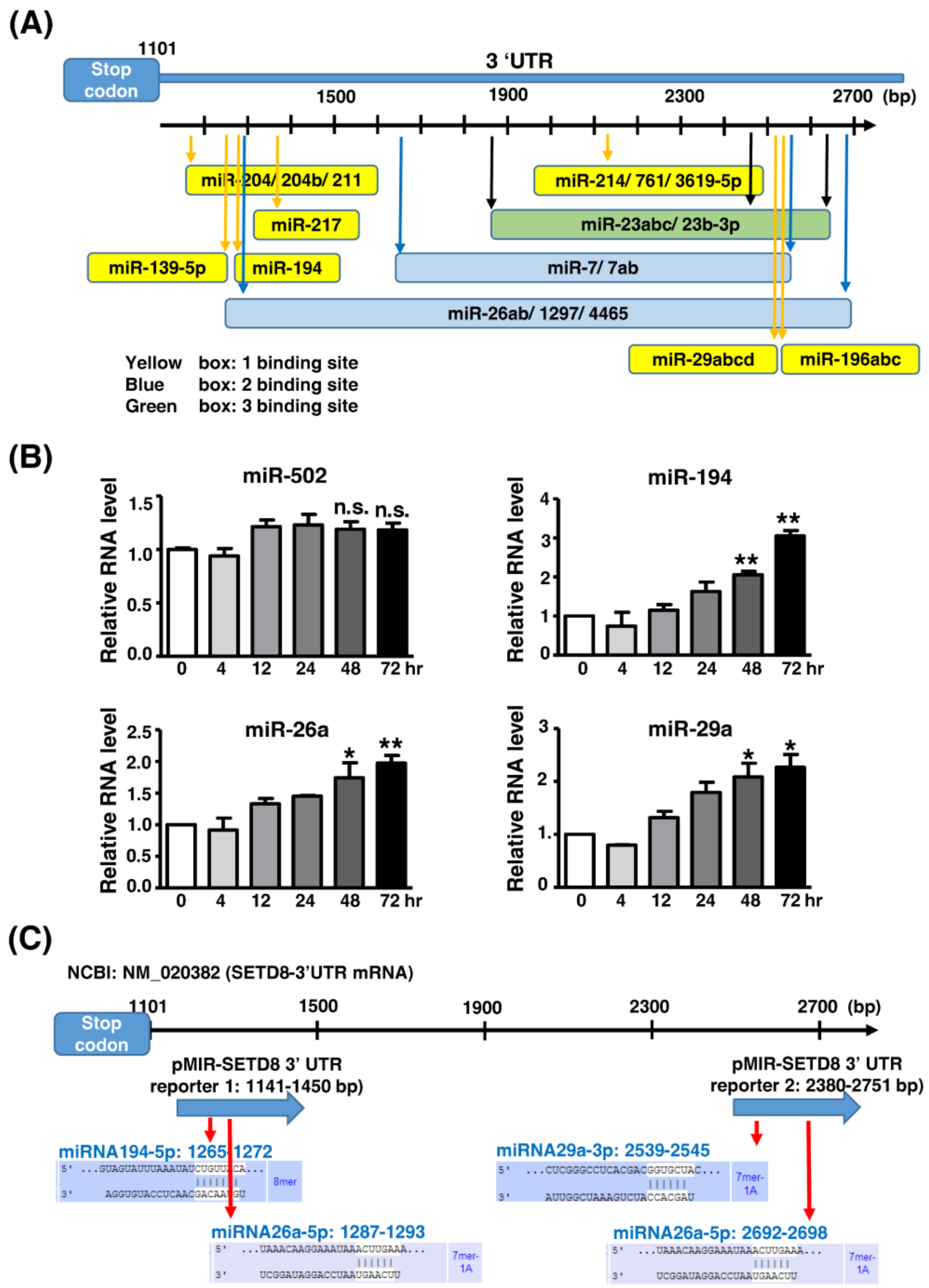
(A)
1101
Stop codon
3 'UTR
1500
1900
2300
2700 (bp)
miR-204/ 204b/ 211
miR-214/ 761/ 3619-5p
miR-217
miR-23abc/ 23b-3p
miR-139-5p
miR-194
miR-7/ 7ab
miR-26ab/ 1297/ 4465
miR-29abcd
miR-196abc
Yellow box: 1 binding site
Blue box: 2 binding site
Green box: 3 binding site
(B)
miR-502
miR-194
miR-26a
miR-29a
Relative RNA level
n.s. n.s.
0 4 12 24 48 72 hr
(C)
NCBI: NM_020382 (SETD8-3'UTR mRNA)
1101
1500
1900
2300
2700 (bp)
Stop codon
pMIR-SETD8 3' UTR reporter 1: 1141-1450 bp)
pMIR-SETD8 3' UTR reporter 2: 2380-2751 bp)
miRNA194-5p: 1265-1272
8mer
miRNA29a-3p: 2539-2545
7mer-1A
miRNA26a-5p: 1287-1293
7mer-1A
miRNA26a-5p: 2692-2698
7mer-1A

Fig. S14

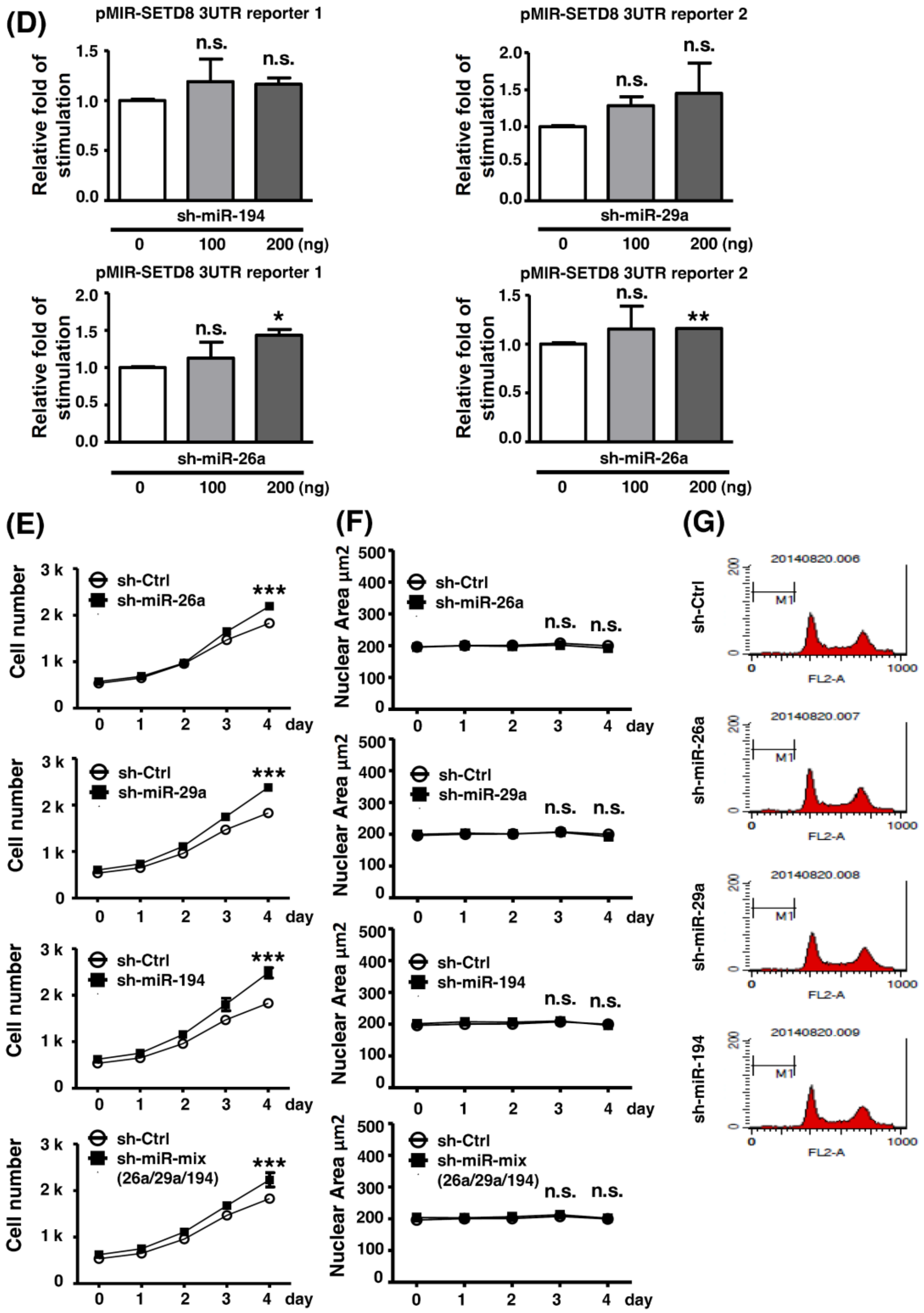
(D)
pMIR-SETD8 3UTR reporter 1
pMIR-SETD8 3UTR reporter 2
Relative fold of stimulation
n.s.
*
**
sh-miR-194
sh-miR-29a
sh-miR-26a
0
100
200 (ng)
(E)
Cell number
sh-Ctrl
sh-miR-26a
sh-miR-29a
sh-miR-194
sh-miR-mix (26a/29a/194)
***
day
(F)
Nuclear Area µm2
(G)
20140820.006
20140820.007
20140820.008
20140820.009
M1
FL2-A

Figure S14. Characterization of possible role of miRNAs in SETD8 expression regulation.

(A) SETD8 3' UTR region contains multiple microRNA binding sites, as predicted by TargetScan. **(B)** Expression patterns of selected miRNAs – miR-26a, miRNA-29a, and miR-194 – in OC3 cells were determined at the indicated time points post-dox treatment, using RT-qPCR. Profiles are shown as mean ± SE of at last three independent experiments, as the 0 hr as the reference. *p < 0.05; **p < 0.01;***p < 0.001. **(C)** Putative miRNA target sites within the SETD8 3' UTR, with the alignment of indicated SETD8 3' UTR/miRNA complements shown below. **(D)** 3' UTR luciferase reporter constructs that contain partial SETD8 3'UTR region (pMIR-SETD8 3UTR-reporter 1 or pMIR-SETD8 3UTR-reporter 2) were co-transfected with the indicated amounts of plasmid encoding ectopic microRNAs into PC3 cells. Reporter activity assay was then performed. Relative levels were normalized to the values of 0 ng group and represent mean ± SE of at last three independent experiments. n.s., not significant; *p < 0.05; **p < 0.01;***p < 0.001. **(E) - (G)** Overexpression of microRNAs 26a, 29a or 194 enhanced cell proliferation, but did not enhance nuclear area enlargement and G2/M arrest. The PC3 cells transfected with the control vector or miRNA-expression plasmids, and subsequently subjected to growth curve analysis **(E)**, nuclear area determination **(F)**, and cell cycle profiling by flow cytometry **(G)**. Values represent mean ± SE of at last three independent experiments and are relative to the Ctrl miRNA. n.s., not significant; ***p < 0.001.

Fig. S15

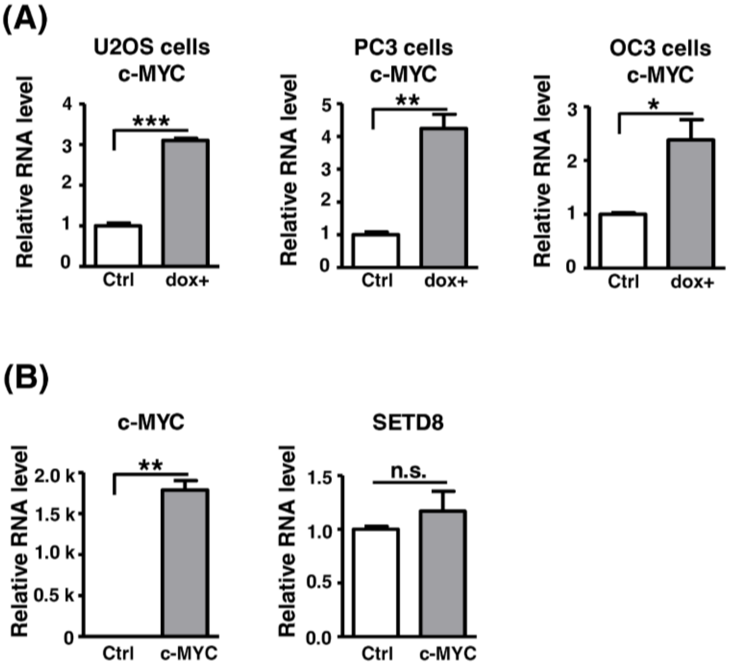

Figure S15. c-MYC is inconsequential in senescence-associated SETD8 down-regulation.

(A) The U2OS, PC3 and OC3 cells were treated with dox to induce senescence, and then examined for c-MYC mRNA levels by RT-qPCR. Mock treatment serves as control. **(B)** Ectopically expressed c-MYC in the PC3 cells. Extent of c-MYC mis-expression and the levels of SETD8 mRNA, were evaluated by RT-qPCR. Relative expression levels, corresponding to mean ± SE of at last three independent experiments, are determined by normalizing to the control transfectants in each experiment. n.s., not significant; *p < 0.05; **p < 0.01.

Fig. S16

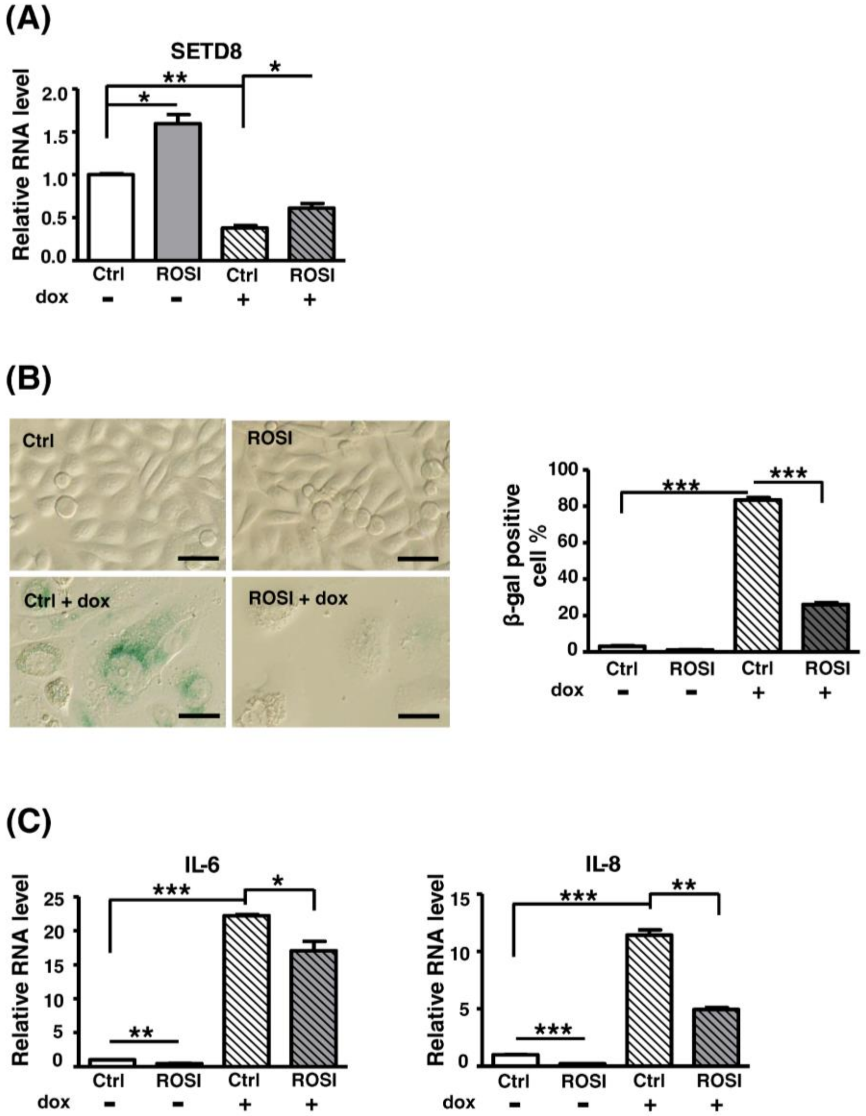

Figure S16. Activation of PPARγ by Rosiglitazone (ROSI) reverses dox-induced senescence.

(A) To examine the role of PPARγ in senescence induction, PC3 cells were co-treated with dox and ROSI, a PPARγ agonist. Extent of SETD8 mRNA up-regulation was assessed by RT-qPCR. Mean ± SE of at last three independent experiments are shown, with the mock treatment group representing the control. **(B) & (C)** The PC3 cells were co-treated with dox and ROSI for 6 days. Senescence induction was determined based on β-gal staining **(B)**. Quantitative representation of the microscopy data is shown in the bar graphs below. Expression of the SASP markers, IL6- and IL-8, was also checked by RT-qPCR **(C)**. Results are normalized to the mock treatment group and shown as mean ± SE of at last three independent experiments**.** *$p < 0.05$; **$p < 0.01$; ***$p < 0.001$.

Fig. S17

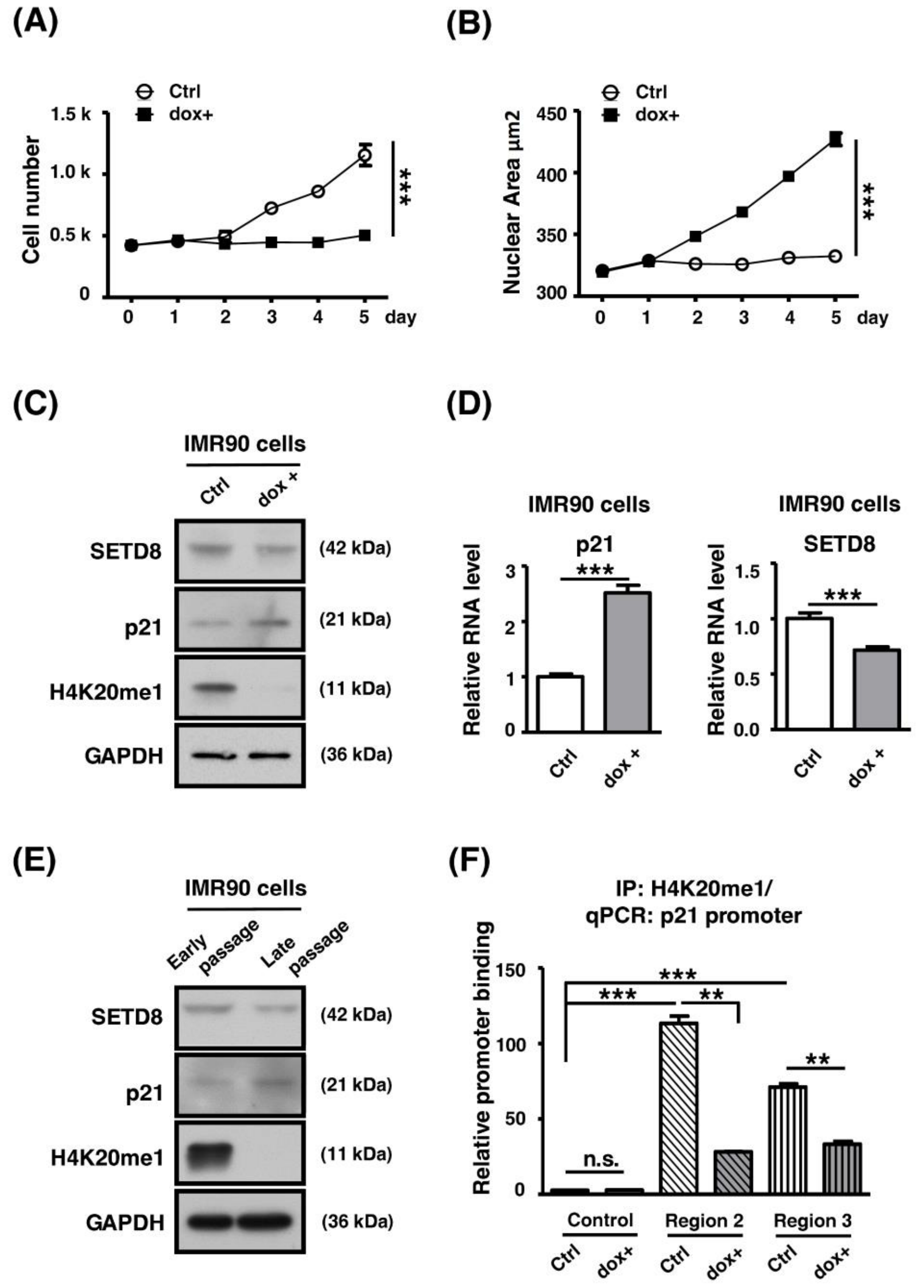
(A)
Ctrl
dox+
Cell number
1.5 k
1.0 k
0.5 k
0
0 1 2 3 4 5 day
***
(B)
Ctrl
dox+
Nuclear Area μm2
450
400
350
300
0 1 2 3 4 5 day
***
(C)
IMR90 cells
Ctrl
dox +
SETD8
(42 kDa)
p21
(21 kDa)
H4K20me1
(11 kDa)
GAPDH
(36 kDa)
(D)
IMR90 cells
p21
Relative RNA level
***
Ctrl
dox +
IMR90 cells
SETD8
Relative RNA level
***
Ctrl
dox +
(E)
IMR90 cells
Early passage
Late passage
SETD8
(42 kDa)
p21
(21 kDa)
H4K20me1
(11 kDa)
GAPDH
(36 kDa)
(F)
IP: H4K20me1/
qPCR: p21 promoter
Relative promoter binding
***
***
**
**
n.s.
Control
Region 2
Region 3
Ctrl
dox+

Figure S17. Senescence-associated expression alteration of SETD8 and H4K20me1 in normal fibroblast cells of IMR90.

(A) to **(E)** The IMR90 cells were treated with 100 ng/ml of dox for 5 days to induce senescence, and then subjected to senescence-related phenotype analyses (see Methods): cell proliferation curve **(A)** and nuclear area determination **(B)**. The cell extracts were prepared for immunoblot analysis of SETD8, p21 and H4K20me1 (GAPDH as the internal control) **(C)**. The mRNA levels of p21 and SETD8 in dox-induced senescence were also verified by RT-qPCR **(D)**. **(E)** Senescence induction by replicative limitation**.** The extracts were prepared from IMR90 in early (24) or late (41) passage for immunoblot analysis of SETD8, p21 and H4K20me1 (GAPDH as the control). **(F)** ChIP analysis of H4K20me1 occupancy of *p21* promoter was performed. Chromatin fragments were isolated from IMR90 cells in the mock (Ctrl) versus dox treatment culture, and immunoprecipitated using control (IgG) or H4K20me1 antibodies. The precipitated DNA fragments were quantitatively analyzed by real-time PCR using the indicated primer pairs, and normalized to the values of IgG. Statistical significance of the indicated comparisons: n.s.: not significant; $^{**}p < 0.01$; $^{***}p < 0.001$.

Fig. S18

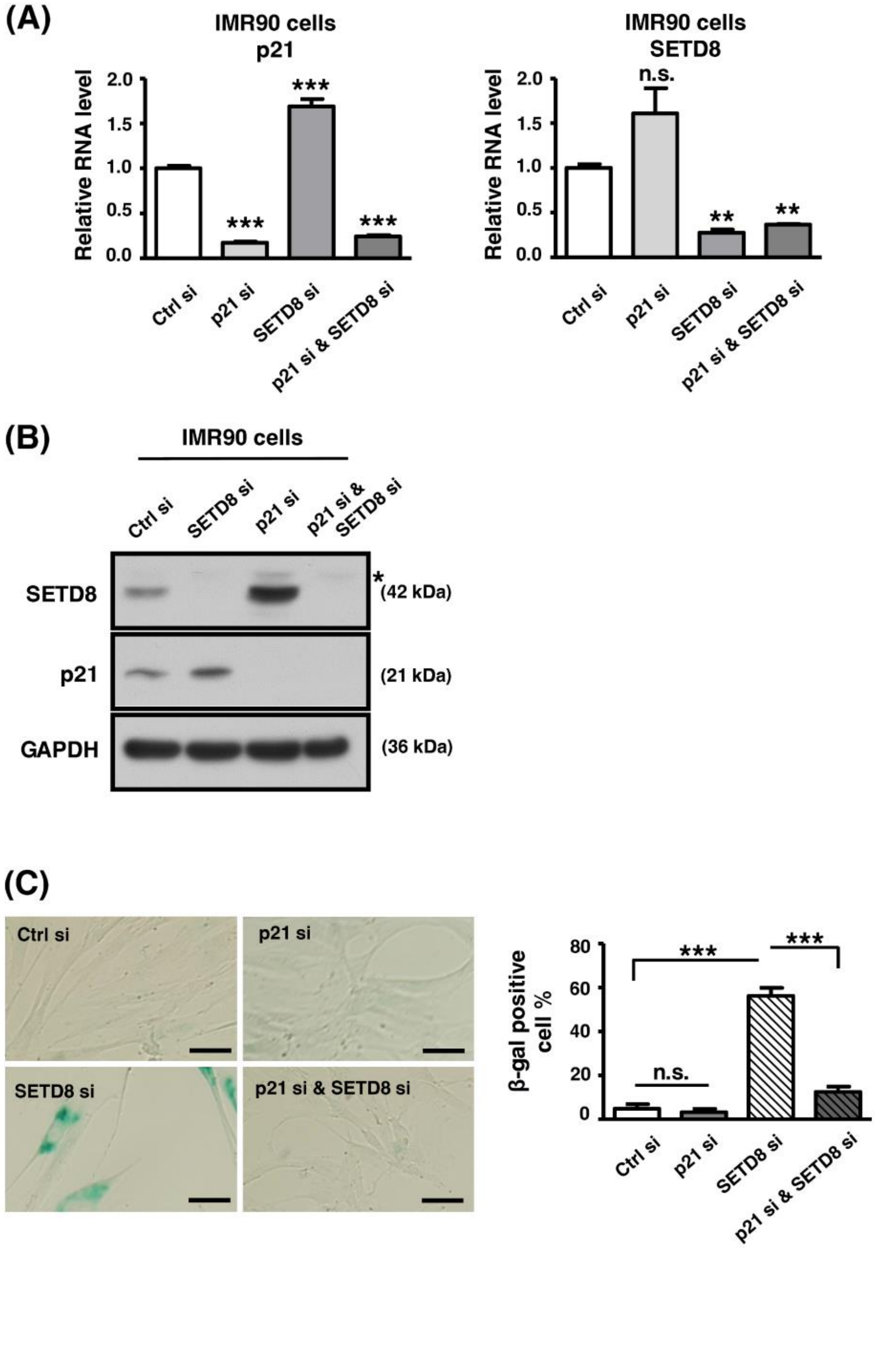
(A)
IMR90 cells
p21
Relative RNA level
2.0
1.5
1.0
0.5
0.0
***
***
***
Ctrl si
p21 si
SETD8 si
p21 si & SETD8 si
IMR90 cells
SETD8
n.s.
**
**
(B)
IMR90 cells
Ctrl si
SETD8 si
p21 si
p21 si & SETD8 si
SETD8
p21
GAPDH
*
(42 kDa)
(21 kDa)
(36 kDa)
(C)
Ctrl si
p21 si
SETD8 si
p21 si & SETD8 si
β-gal positive cell %
80
60
40
20
0
n.s.
***
***

Fig. S18

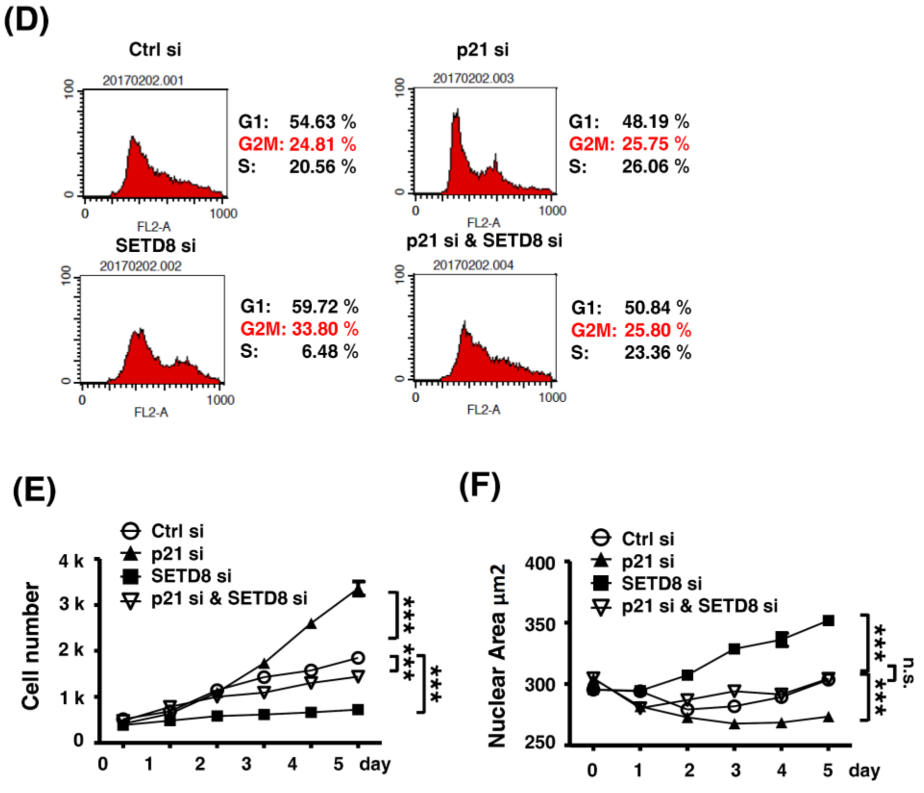

Figure S18. Knockdown of p21 alleviates the senescent state of the SETD8-depleted IMR90 cells.

(A) to **(F)** IMR90 cells were transfected with SETD8-targeting siRNAs, p21-targeting siRNA, or both for 3 days. Extent of *p21* or *SETD8* knockdown in IMR90 cells was assessed by real-time RT-PCR **(A)** or immunoblotting **(B)**. Normalized expression levels are shown in **(A)**, as indicated (GAPDH as the internal control). The asterisk in **(B)** denotes a non-specific signal in the anti-SETD8 blot. Senescence-related characterization was conducted as above, on the basis of β-gal+ staining (scale bar = 50 μm) **(C)**, flow cytometry-based cell cycle profiling **(D)**, cell proliferation rate **(E)**, and nuclear area measurement **(F)**. All results are shown as mean ± SE of at last three independent experiments (n.s.: not significant; $**p < 0.01$; $***p < 0.001$).

Fig. S19

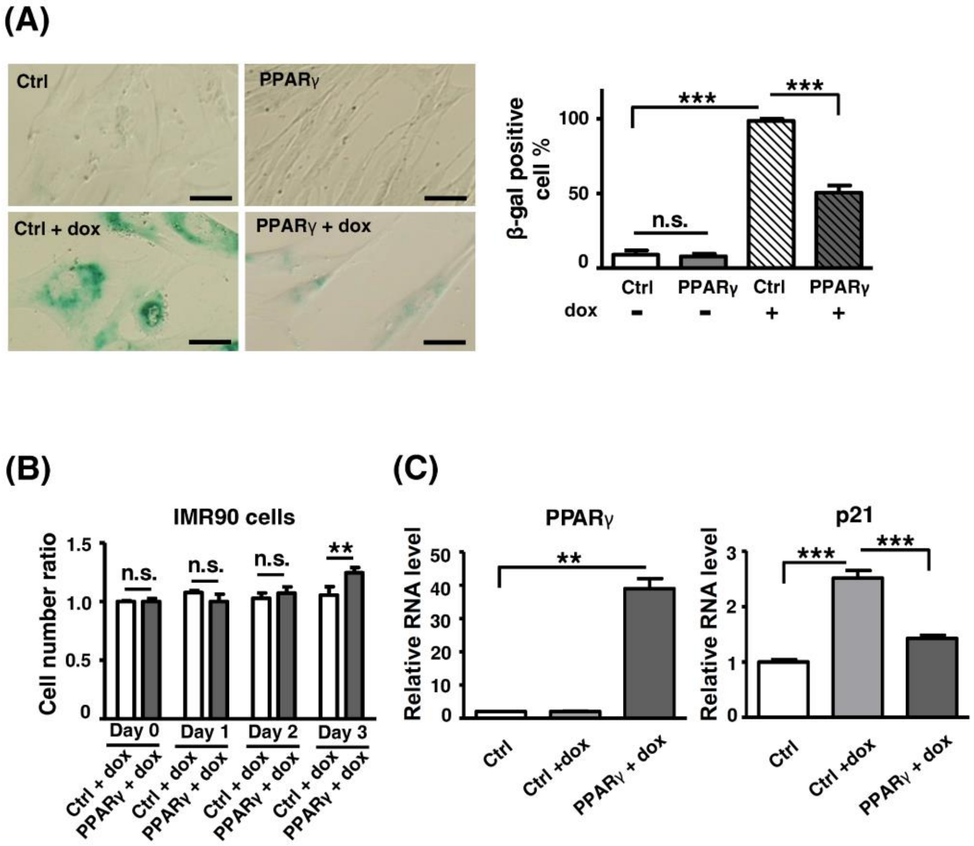

Figure S19. Negative regulation of cellular senescence by PPARγ in IMR90 cells. Effect of PPARγ over-expression in counteracting cellular senescence. Upon transfection with the Ctrl or PPARγ-expressing plasmids, IMR90 cells were treated with dox for up to 6 days to induce cellular senescence. Cells were then subjected to β-gal staining (scale bar = 50 μm; bar graph on the right shows the percentage of β-gal+ cells in the indicated culture) **(A)** or cell growth analysis **(B)**. The *PPARγ* (left) or *p21* (right) mRNA expression level was analyzed by real-time RT-PCR **(C)**. All results are shown as mean ± SE of at least three independent experiments (n.s.: not significant; $^{**}p < 0.01$; $^{***}p < 0.001$).

Fig. S20

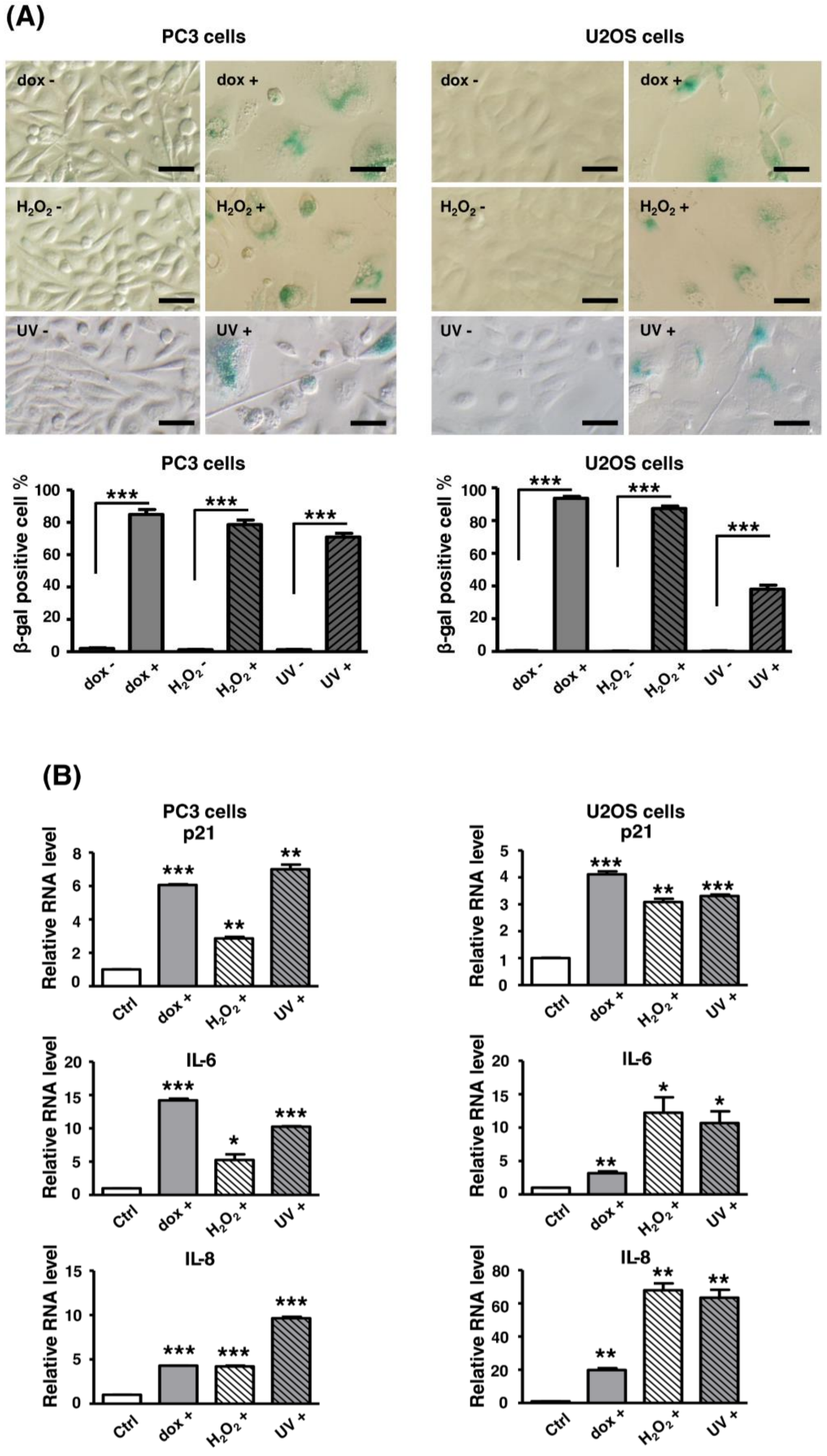
(A)
PC3 cells
U2OS cells
dox -
dox +
H2O2 -
H2O2 +
UV -
UV +
β-gal positive cell %
***
(B)
p21
IL-6
IL-8
Relative RNA level
Ctrl
*
**

Fig. S20

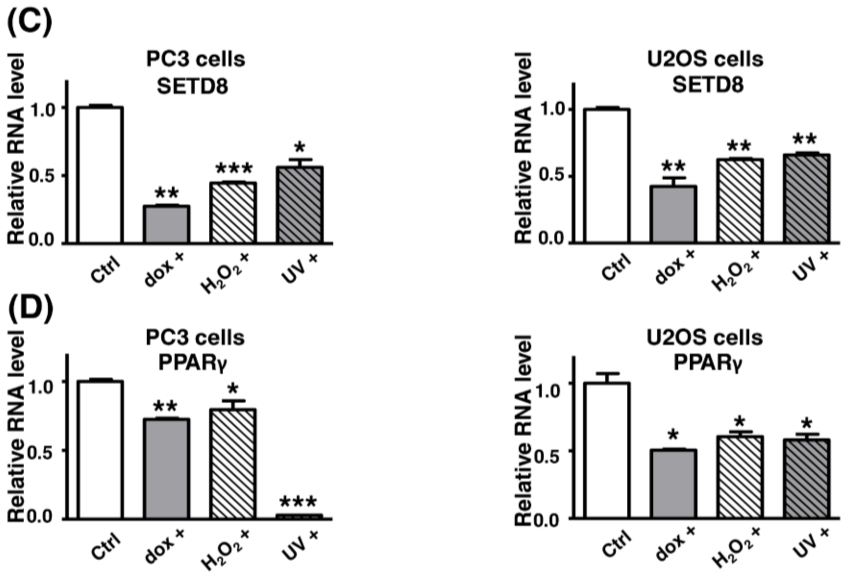

Figure S20. SETD8 down-regulation in multiple DNA damage factors-induced cellular senescence.

(A) – (D) To induce cellular senescence via distinct sources of DNA damage, both of U2OS and PC3 cells were treated with dox, H_2O_2, or UV for 6 days. Senescence-related phenotype analyses were then performed using β-gal staining (scale bar = 50 μm for the images, with bar graph below depicting quantitative results) **(A)** and expression profiling of senescence-associated genes – p21, IL-6, and IL-8 – by RT-qPCR **(B)**. Expression alteration of SETD8 **(C)** and PPARγ **(D)** was also characterized by RT-qPCR. All quantitative results shown are presented as mean ± SE of at last three independent experiments and relative to the mock treatment (Ctrl) (n.s.: not significant; $*p < 0.05$; $**p < 0.01$; $***p < 0.001$).

Fig. S21

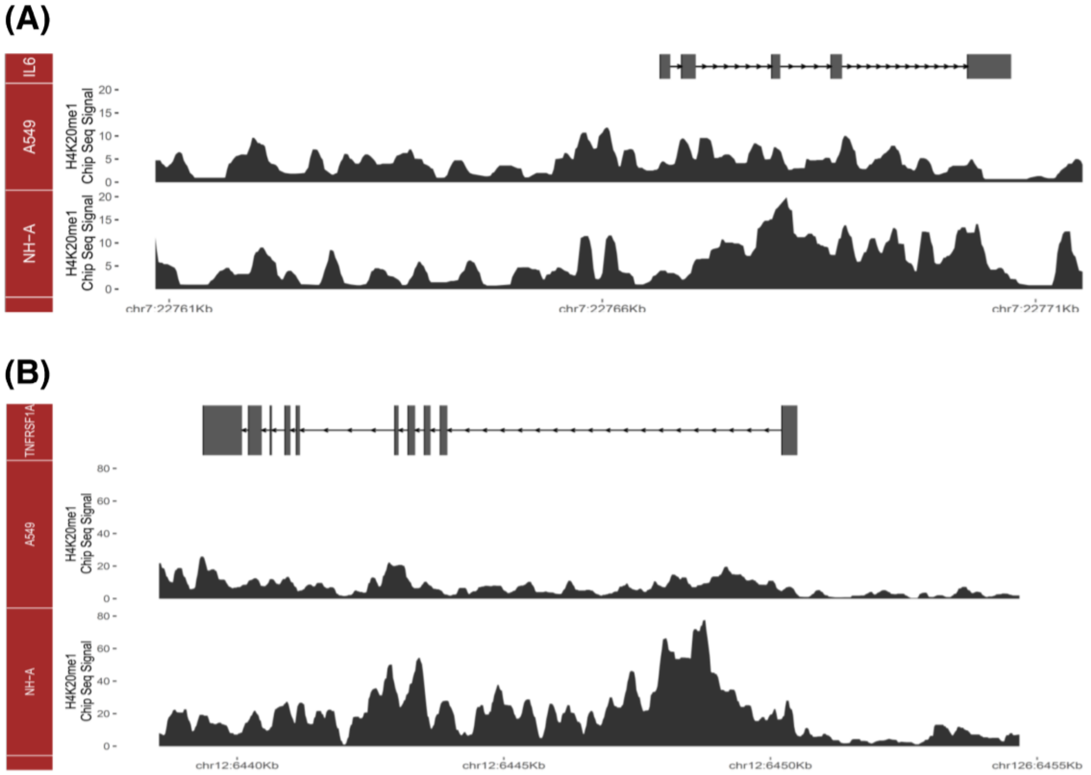

Figure S21. H4K20me1 distribution in SASP gene regions.
Chromatograms depicting positional occupancy of the H4K20me1 mark in the *IL-6* **(A)** and *TNFRSF1A* **(B)** gene loci. The ENCODE annotated data for the selected cell lines are shown based on UCSC genome browser-based representation.

Fig. S22

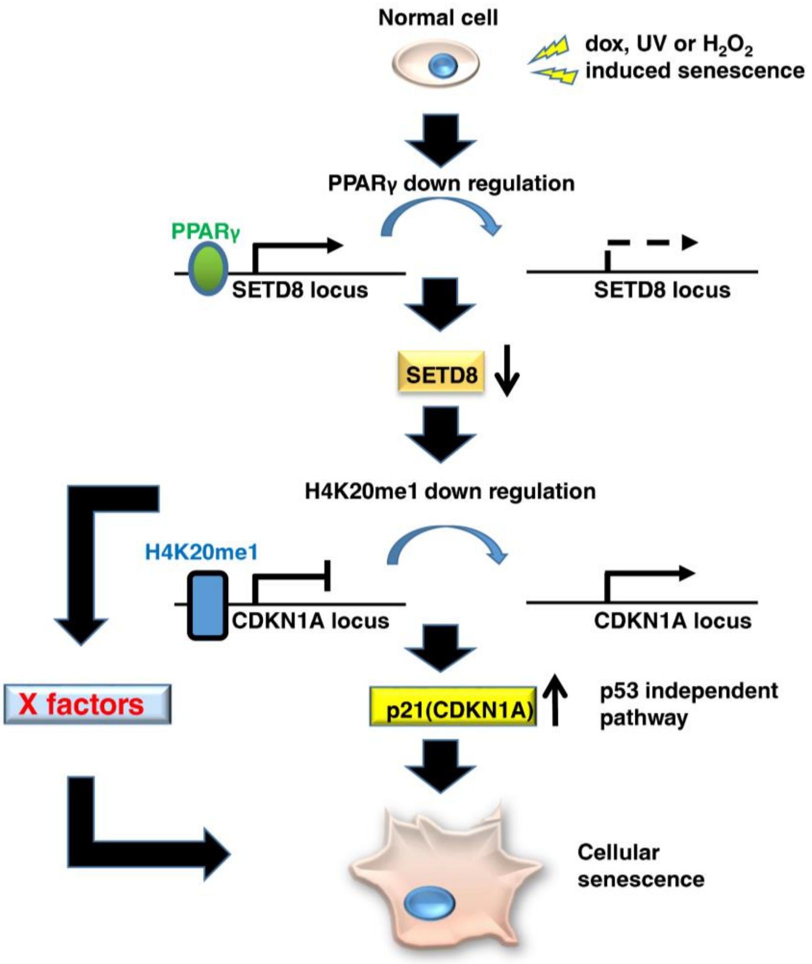

Figure S22. Schematic model for the functional implication of the PPARγ-SETD8-H4K20me1 pathway in cellular senescence.

Our current report delineated a novel PPARγ-SETD8 pathway that impinges on the *p21* gene and its senescence inducing function. In the presence of genome insulting stresses, this checkpoint mechanism is down-regulated, leading to de-repression of *p21* and the consequence cellular senescence induction. While our results demonstrated that this regulatory pathway is independent of p53, they do not exclude the existence of additional factors with cooperative action. See text for further discussion.
